# Beyond Insulin Resistance: Exploring the Centrality of the Gut–Liver Axis in Mediating Immunometabolic Dysregulation Driving Hepatocellular Carcinoma in MASLD and Diabetes

**DOI:** 10.3390/cancers18081316

**Published:** 2026-04-21

**Authors:** Mario Romeo, Claudio Basile, Giuseppina Martinelli, Fiammetta Di Nardo, Carmine Napolitano, Alessia De Gregorio, Paolo Vaia, Luigi Di Puorto, Mattia Indipendente, Alessandro Federico, Marcello Dallio

**Affiliations:** Hepatogastroenterology Division, Department of Precision Medicine, University of Campania Luigi Vanvitelli, Piazza Miraglia 2, 80138 Naples, Italy; mario.romeo@unicampania.it (M.R.); claudio.basile@unicampania.it (C.B.); giuseppina.martinelli@studenti.unicampania.it (G.M.); fiammetta.dinardo@unicampania.it (F.D.N.); carmine.napolitano@unicampania.it (C.N.); alessia.degregorio2@studenti.unicampania.it (A.D.G.); paolo.vaia@unicampania.it (P.V.); luigi.dipuorto2@studenti.unicampania.it (L.D.P.); mattia.indipendente@studenti.unicampania.it (M.I.); alessandro.federico@unicampania.it (A.F.)

**Keywords:** precision medicine, gut–liver axis, hepatocellular carcinoma

## Abstract

Hepatocellular carcinoma (HCC) represents a major global health challenge and the third leading cause of cancer-related mortality worldwide. Its epidemiological burden is rapidly increasing, largely driven by the rising prevalence of metabolic dysfunction-associated steatotic liver disease (MASLD), which is now recognized as the most common chronic liver disease globally. Notably, MASLD frequently coexists with type 2 diabetes mellitus (T2DM), sharing several features, including the interplay of common genetic, metabolic, and environmental factors, thus contributing to a complex multifactorial pathogenesis. Relevantly, patients affected by both conditions represent a subgroup at particularly high risk of liver disease progression and hepatocarcinogenesis. In this population, metabolic and inflammatory disturbances act synergistically to create a pro-tumorigenic hepatic environment where insulin resistance (IR) plays a crucial role, by driving hepatic lipotoxicity, mitochondrial dysfunction, and inflammatory signaling with oxidative stress, thereby establishing a permissive environment for worsening steatosis and malignant transformation. Increasing evidence supports the concept of MASLD as a multisystem disorder reflecting the systemic nature of metabolic dysfunction. Within this framework, beyond IR, extrahepatic factors have also emerged as important contributors to steatosis progression, worsening of T2DM, and modulation of HCC risk. In particular, the gut–liver axis has gained recognition as a key regulator of hepatic homeostasis, integrating signals from the intestinal microbiota, immune responses, and metabolic pathways. Dysregulation of this crosstalk promotes systemic inflammation and metabolic imbalance, exacerbating IR and fostering a pro-oncogenic hepatic environment. This review examines the interconnected metabolic and immune mechanisms linking IR and gut–liver axis dysfunction to HCC development in patients with MASLD and T2DM, highlighting their implications for risk stratification and precision-based therapeutic strategies.

## Simple Summary

Liver cancer is a leading cause of cancer-related death worldwide, and its incidence is increasing, largely due to the growing prevalence of metabolic liver disease and type 2 diabetes. These conditions are closely linked and can promote liver damage and cancer development even before advanced liver scarring occurs. Traditionally, research has focused on insulin resistance as the main driver of this process. However, recent evidence highlights the important role of interactions between the gut and the liver, where changes in intestinal bacteria and increased gut permeability can trigger inflammation and worsen metabolic imbalance. This review explores how metabolic alterations and immune dysfunction interact to promote liver cancer, with a particular focus on the gut–liver axis. Understanding these mechanisms may help identify patients at higher risk and support the development of more personalized strategies for prevention, early detection, and treatment.

## 1. Introduction

Hepatocellular carcinoma (HCC) represents a major global health challenge and currently ranks as the third leading cause of cancer-related mortality worldwide [1,2]. Its epidemiological burden is steadily increasing, reflecting profound shifts in the global landscape of chronic liver diseases [1,2], where metabolic dysfunction-associated steatotic liver disease (MASLD) is emerging as the most prevalent condition worldwide, affecting approximately one quarter of the global population [3,4]. Recent epidemiological analyses estimate a global prevalence of MASLD of approximately 38% in the adult population, with projections indicating a possible increase to over 55% in the next decade [3]. In this context, MASLD configures a dramatic picture where both liver disease progression and HCC occurrence are promoted [3] in a *sui generis* scenario where primary hepatic cancer onset has been reported even in the absence of advanced fibrosis (AF), alarmingly suggesting the independence of carcinogenetic mechanisms from worsening of fibrosis [3,5].

Notably, MASLD-related HCC displays distinctive features compared with viral- or alcohol-related liver disease. A growing body of evidence indicates that HCC can develop in MASLD patients even in the pre-cirrhotic stage, suggesting that metabolic and inflammatory carcinogenic pathways may operate independently of the classical fibrosis-driven model of hepatocarcinogenesis [6]. This paradigm shift has major clinical implications, as current surveillance strategies are largely restricted to cirrhotic populations, potentially leading to under-recognition of cancer risk in a substantial proportion of MASLD patients.

Importantly, the oncological burden of MASLD is not limited to HCC, since emerging evidence has demonstrated a significant association between steatotic liver disease and primary liver cancer, including intrahepatic cholangiocarcinoma (iCCA), suggesting that shared metabolic, inflammatory, and microenvironmental alterations may contribute to carcinogenesis [7]. This observation expands the spectrum of MASLD-related liver malignancies and further supports the role of metabolic dysfunction (MD) as a unifying driver of hepatic cancer development.

From a clinical point of view, this evidence reveals the Achilles’ heel of current screening and surveillance strategies, which remain largely limited to patients with AF [6], and reinforces the need to conceive MASLD as the hepatic expression of a systemic (MD).

In line with this, parallel to the expansion of MASLD, the global prevalence of type 2 diabetes mellitus (T2DM) has reached epidemic proportions, representing one of the most pressing metabolic health challenges of the twenty-first century [8].

Currently, T2DM is no longer conceived as a mere disorder of glucose homeostasis but rather as a complex systemic metabolic disease characterized by widespread alterations in insulin signaling, chronic low-grade inflammation, and profound MD.

Importantly, MASLD and T2DM frequently coexist and share a complex bidirectional relationship [9]. Available evidence indicates an association between MASLD and T2DM in a scenario where T2DM significantly increases the risk of MASLD progression, whereas MASLD itself predisposes individuals to incident T2DM [9]. In relation to this, a recent large-scale meta-analysis has reaffirmed an elevated global prevalence of T2DM in patients with hepatic steatosis associated with MD [10]. Moreover, emerging findings consistently demonstrate that the coexistence of MASLD and diabetes identifies a subgroup of patients at particularly high risk of advanced liver disease and hepatocarcinogenesis [9,11]. In line with this, a large meta-analysis by Huang et al. confirmed a significantly higher risk of developing HCC in patients with MASLD affected by T2DM compared with nondiabetic individuals [9,11].

From an epidemiological perspective, the clinical burden associated with MASLD and T2DM extends far beyond their high prevalence. MASLD is currently one of the leading causes of cirrhosis and liver-related morbidity worldwide, with progressive forms of disease expected to become a major indication for liver transplantation in the coming years [12]. In parallel, T2DM has been consistently associated with a significantly increased risk of AF, cirrhosis, and HCC [10,12]. Beyond clinical outcomes, this scenario also entails substantial socioeconomic consequences, including increasing healthcare expenditures, reduced quality of life, loss of productivity, and growing pressure on healthcare systems worldwide [12]. Relevantly, when MASLD and T2DM coexist, their detrimental effects appear to be synergistic, identifying a particularly vulnerable population with markedly higher rates of hepatic decompensation, cancer development, and liver-related mortality [10,12].

In light of these epidemiological observations, increasing attention has been directed toward the shared pathogenic mechanisms underlying MASLD and T2DM [9,11]. Notably, both conditions arise from overlapping pathogenic mechanisms, including genetic susceptibility, metabolic dysregulation, mitochondrial dysfunction, oxidative stress, adipose tissue inflammation, and immune remodeling [9,11]. Central to this MASLD-T2DM shared pathogenic network is insulin resistance (IR), which represents a pivotal metabolic disturbance linking hepatic steatosis, lipotoxicity, mitochondrial dysfunction, oxidative stress, and chronic inflammation [4].

IR primarily promotes hepatic accumulation of free fatty acids (FFAs) as a crucial event in the pathogenesis of steatosis [4,13], where lipid overload and impaired mitochondrial function determine production of reactive oxygen species (ROS), ultimately resulting in oxidative stress [4,14,15]. Notably, through these mechanisms, IR contributes to the initiation and progression of MASLD, as well as to the establishment of a pro-oncogenic hepatic microenvironment [16].

Considering this, for decades, IR has represented the exclusive and main pathogenetic driver linking liver disease, T2DM, and HCC progression [16]. Intriguingly, in recent years, in line with emerging evidence suggesting MASLD as a multisystem disease reflecting the systemic nature of MD [17], accumulating findings have revealed the relevance of extrahepatic pathogenetic factors in contributing to the worsening of steatosis, progression of T2DM, and the modulation of HCC risk [1,18].

In particular, within this evolving framework, the gut–liver axis has emerged as a central regulator of hepatic homeostasis [19], and gut-derived molecules have been shown to be crucial regulators of systemic metabolism and immune activation status [20]. Interestingly, in this context, IR and gut–liver axis dysfunction have progressively emerged as interconnected drivers of hepatocarcinogenesis rather than independent processes [21]. In this sense, increasing evidence indicates that gut-derived signals, including microbial products, actively contribute to the development and maintenance of IR [22].

**Figure 1:**
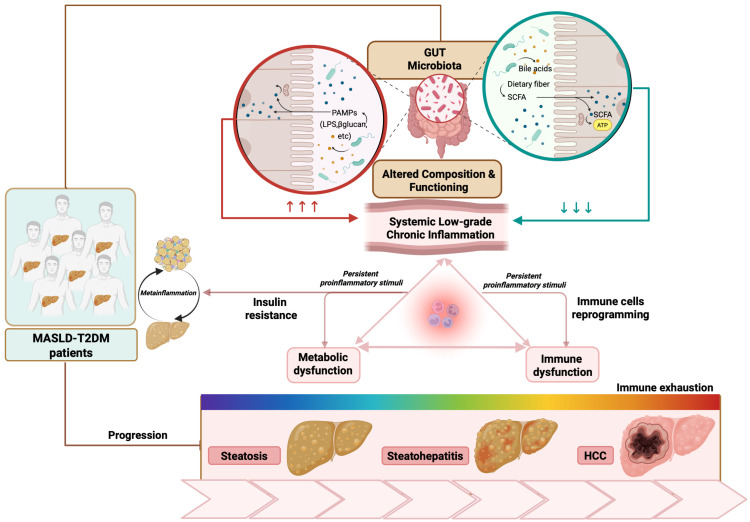
Gut–liver axis dysregulation controls the immunometabolic crosstalk driving disease progression and hepatocancerogenesis in the setting of MASLD-T2DM. MASLD: Metabolic dysfunction-associated steatotic liver disease. T2DM: Type 2 diabetes mellitus. SCFAs: short-chain fatty acids. HCC: hepatocellular carcinoma. LPS: lipopolysaccharide. PAMPs: Pathogen-Associated Molecular Patterns; ↑↑↑: increase; ↓↓↓: decrease. Created in BioRender. Romeo, M. (2026) https://BioRender.com/z7jgub6 (accessed on 14 April 2026).

At the same time, IR itself promotes alterations in gut permeability and microbiota composition. This bidirectional interplay creates a self-reinforcing loop that amplifies MD, chronic inflammation, and immune dysregulation, ultimately fostering a pro-tumorigenic hepatic environment [21,23]. Taken together, this evidence highlights the relevance of investigating the role of the gut–liver axis as a key mechanistic interface linking metabolic, immune dysregulation, and liver carcinogenesis, configuring a scenario where the alterations in microbiota composition and functioning can fuel systemic inflammation, ultimately worsening IR and MD (Figure 1) [1].

In the evolving landscape of modern hepatology, understanding these interactions may help identify novel actionable targets for risk stratification, prevention, and precision-based therapeutic strategies.

This narrative review was based on a non-systematic literature search performed in PubMed/MEDLINE, Scopus, and Web of Science up to January 2026. The search included combinations of the following keywords: “MASLD”, “MAFLD”, “type 2 diabetes”, “insulin resistance”, “gut–liver axis”, “microbiota”, “hepatocellular carcinoma”, “immune dysfunction”, and “immune checkpoint inhibitors”.

Priority was given to original studies, landmark mechanistic investigations, high-quality clinical studies, and recent review articles considered relevant to the scope of the manuscript. Additional references were identified through manual screening of bibliographies from selected articles.

Although several reviews have separately addressed the roles of IR, MASLD progression, gut microbiota alterations, or immune mechanisms in hepatocarcinogenesis, an integrated framework connecting these processes in the specific setting of MASLD and T2DM remains lacking. The novelty of the present review lies in its multidimensional perspective, which jointly examines MD, gut–liver axis dysregulation, and immune remodeling as interconnected drivers of HCC development. It should be recognized that some of the mechanisms discussed in this review are not exclusive to MASLD or T2DM, but overlap with pathways commonly associated with obesity, which frequently coexist with both conditions [24]. In particular, adipose tissue inflammation, altered adipokine secretion, systemic low-grade inflammation, and worsening IR are strongly influenced by excess adiposity and may indirectly contribute to liver injury and hepatocarcinogenesis [25]. However, accumulating evidence suggests that MASLD and T2DM also exert pathogenic effects beyond obesity itself, and these include hepatic IR, glucotoxicity, ectopic lipid deposition, mitochondrial dysfunction, gut–liver axis alterations, and diabetes-related immune dysregulation, which may occur even in the absence of overt obesity [26,27]. The existence of lean MASLD further supports the concept that obesity amplifies, but does not fully explain, the biological link between MASLD, T2DM, and HCC [17,26].

Finally, we specifically focus on the coexistence of MASLD and T2DM as a high-risk clinical phenotype and discuss the potential translational implications of these mechanisms for risk stratification and precision-based therapeutic strategies.

## 2. Insulin Resistance Promotes Hepatocarcinogenesis in MASLD-T2DM

### 2.1. The Pleiotropic Role of the Insulin/Insulin-like Growth Factor-1 Axis in Hepatocarcinogenesis

In the heterogeneous landscape of hepatocancerogenesis, the IR-related hyperinsulinemia determines the activation of crucial signaling mechanisms controlling pro-proliferative and pro-survival effects, which significantly contribute to HCC progression in the MASLD–T2DM setting [9,28,29]. In particular, the insulin/insulin-like growth factor-1 (IGF-1) axis has been described as being crucially involved in the regulation of cell proliferation and survival in liver cancer cells [9,28,29]. Interestingly, in patients with HCC, serum levels of IGF-1 are substantially reduced, reflecting damage to hepatic parenchyma and decreased growth hormone receptor expression [30]. However, this reduction in circulating IGF-1 is contrasted by increased expression of IGF-1 receptor (IGF-1R) in HCC tissue compared to normal liver [30]. Consistently, among the most significant alterations initially found in hepatoma cell lines is the increased expression of the IGF-1R, confirming its role as a key element in neoplastic transformation processes and disease progression [9,31]. Subsequently, further experimental evidence has reinforced the biological role of IGF1R dysregulation in HCC, revealing a significant overexpression of IGF1R in tumor tissues compared to non-neoplastic liver tissue, and high levels of IGF1R expression are associated with a worse prognosis in patients with HCC [32]. Consistently, IGF1R is universally recognized as a key component of the IGF axis and is currently considered to be implicated in HCC tumor progression, therapeutic resistance, and metastasis [32,33,34,35,36].

In the setting of MASLD and MASLD-T2DM, the compensatory hyperinsulinemia associated with IR has been reported to be related to increased hepatic IGF-1 levels, resulting in the enhanced activation of the IGF1R and amplification of biological programs compatible with tumor progression, including proliferation, invasiveness, and reduced apoptosis [9,37].

In this context, IGF-1R shifts from membrane localization to predominantly nuclear localization in tumor tissue, suggesting a role in nuclear signaling that contributes to malignant transformation [38]. At the nuclear level, IGF-1R drives HCC cell proliferation through several intracellular pathways. IGF-1R silencing reduces HCC cell proliferation and invasiveness while enhancing apoptotic processes through inhibition of the phosphatidylinositol-3-kinase (PI3K)/protein kinase B (AKT) signaling pathway [39]. This PI3K/AKT activation represents a critical mechanism through which IGF-1R mediates cancer cell survival, as IGF-1R-induced signaling leads to AKT phosphorylation and downstream survival signals [39].

Further mechanistic evidence has reinforced the functional role of the IGF1/IGF1R axis in regulating the biology of hepatic cancer stem cells. Stimulation with IGF1 has been associated with increased expression of stemness-related genes and enhanced sphere-forming capacity, while overexpression of IGF1R has resulted in a significant increase in the stem cell markers Octamer-binding transcription factor 4 (Oct4) and Nanog homeobox transcription factor (Nanog) [32].

Besides this, to gain deeper insight into the molecular complexity of this signaling cascade in HCC, recent research has documented significant alterations in the IGF axis, which contribute to the molecular pathogenesis of HCC [9]. Specifically, mechanisms such as autocrine IGF production, dysregulation of IGF-binding proteins (IGFBPs), and enhanced activity of IGFBP-specific proteases have been described [9,31].

Emerging evidence highlights the cruciality of the autocrine IGF-1 axis in HCC progression. Within the tumor microenvironment, cancer-associated fibroblasts (CAFs) have been shown to promote HCC cell proliferation and inhibit apoptosis through the secretion of interleukin (IL-)6 (IL-6). IL-6, in turn, induces autocrine IGF-1 production in tumor cells [40]. Activation of this IL-6–IGF-1 signaling axis leads to stimulation of the IGF-1R, triggering downstream oncogenic pathways, including Signal Transducer and Activator of Transcription 3 (STAT3) phosphorylation and AKT signaling via the Extracellular Signal-Regulated Kinase (ERK) pathway [40]. Importantly, pharmacological inhibition of IGF-1R through the selective inhibitor NT157 has been shown to suppress these pro-tumorigenic effects and enhance the sensitivity of HCC cells to sorafenib, highlighting the potential translational relevance of targeting the IGF signaling pathway in HCC [40].

In this sense, IGF-1R plays a critical role in sorafenib resistance in HCC. A positive feedback loop between Yes-associated protein (YAP) and IGF-1R drives sorafenib resistance, with IGF-1/2 treatment enhancing YAP nuclear translocation, which in turn upregulates IGF-1R expression [41]. Consistently, blocking YAP with the specific inhibitor verteporfin significantly increases HCC cell sensitivity to sorafenib, suggesting that disrupting the YAP-IGF-1R signaling loop can overcome drug resistance [41]. Interestingly, niclosamide has been revealed to potentially enhance sorafenib sensitivity in sorafenib-resistant HCC cells by modulating IGF-1R expression and suppressing stemness-related properties [42]. This combination approach reduces metabolic adaptations that support drug resistance and promotes apoptosis in both cell culture and xenograft models [42].

In this sense, in addition to their direct effects on cancer cells, metabolic signaling pathways can also influence the antitumor immune response. In this context, intervention on metabolic checkpoints, including mammalian Target of Rapamycin (mTOR) and Hypoxia-Inducible Factor 1α (HIF-1α), can modulate the differentiation and functionality of T lymphocytes, opening up the possibility of synergistic integration with immunotherapies based on immune checkpoint inhibitors (ICIs) [9,43].

In parallel with these emerging insights, in the MASLD–T2DM context, the IGF-1 pathway has also been implicated in HCC development in diabetic patients through the activation of autophagic processes [9,44]. Autophagy is an adaptive mechanism that allows cancer cells to survive in unfavorable microenvironmental conditions, such as hypoxia or nutrient deprivation, which are typically found in growing solid tumors [9,45].

In line with these data, pharmacological inhibition studies have shown that blocking IGF1R via Picropodophyllin (PPP) reduces the expansion of liver cancer stem cells (CSCs) and is associated with a decrease in tumor growth and metastatic spread, suggesting a possible translational relevance of targeting this pathway [32,46,47,48,49,50].

Altogether, this evidence reinforces the crucial role of IR in contributing to HCC onset and progression in the MASLD–T2DM setting, both directly, through the hyperinsulinemia-determined activation of signaling mechanisms controlling pro-proliferative and pro-survival effects, and indirectly, through compensatory hyperglycemia-related effects, which are detailed in the next dedicated subparagraph [9,28,29].

### 2.2. IR–Related Hyperglycemia as a “Classic” Cancer-Promoting Signaling Method in MASLD-T2DM: An Overview of the Role of Advanced Glycation End-Products in Hepatic Carcinogenesis

In the specific context of MASLD–T2DM, IR has been recognized as a central player involved in promoting liver carcinogenesis [9], as also supported by observational research documenting an association between the presence of T2DM and an increased risk of liver cancer in patients showing hepatic steatosis and the features of MD [9].

In particular, in the MASLD phenotype associated with T2DM, uncontrolled hyperglycemia leads to the production of Advanced Glycation End-products (AGEs), representing the pathogenetic core of the main diabetic hepatic and extrahepatic complications [51,52,53].

As is known, AGEs are formed through the Maillard reaction, in which reducing sugars react non-enzymatically with amino groups of proteins, lipids, and nucleic acids, passing through the formation of a Schiff base, an Amadori rearrangement, and subsequent oxidative modifications [51,54,55,56].

Considering that higher plasma glucose levels typically promote an enhanced protein glycation [57], in the MASLD-T2DM setting, chronic IR-related hyperglycemia constantly promotes the progressive accumulation of AGEs [57,58].

Interestingly, growing evidence suggests that AGEs, through their interaction with Receptor for Advanced Glycation End-products (RAGE), are crucial molecules involved in various types of neoplasms, promoting tumor growth, cell migration, and metastatic processes [57]. In vitro studies have shown that exposure to AGEs promotes growth, migration, and invasiveness in cancer cell lines [57]. In line with these findings, an exalted presence of AGEs, particularly Nε-(Carboxymethyl)lysine (CML) and argpyrimidine, has been demonstrated in various human tumors, including breast and colon adenocarcinomas [57,59]. Moreover, several population studies have shown that high concentrations of AGEs in the blood—resulting from diet or pathological conditions including T2DM—are associated with an increased risk of advanced breast cancer [60], prostate cancer [61], and HCC [62].

In this scenario, the liver plays a key role in the catabolism and removal of AGEs through sinusoidal endothelial cells (SECs) and Kupffer cells [63].

However, in the presence of liver dysfunction, as well as when they are overproduced, these “glycotoxins” accumulate, promoting the creation of a pro-tumorigenic environment [63].

In particular, the toxic AGEs (TAGE), which are glyceraldehyde-derived AGEs, have been specifically associated with MASLD-related HCC [64], and they have been reported to mainly deposit in the extracellular matrix (ECM), where they alter tissue mechanics and promote cancer progression [65]. By accumulating at this site, AGEs alter collagen architecture and enhance ECM viscoelasticity, ultimately facilitating hepatocyte proliferation and invasion through integrin-mediated mechanotransductive pathways [65].

This pathogenic process is further amplified by α-dicarbonyl compounds, which are highly reactive metabolic byproducts closely linked to overweight-induced metabolic disorders and AGE formation in patients with MASLD and HCC [66].

In this sense, hepatic cells appear significantly vulnerable to carbonyl stress due to reactive carbonyl species, including α-dicarbonyls, whose presence in the setting of MASLD is associated with higher hepatic RAGE expression levels [63,66].

Interestingly, RAGE has been found to be overexpressed in primary HCC compared to adjacent non-cancerous liver tissue [67], and the interactions between RAGE and its ligands, including HMGB1 and S100A4, further increase RAGE expression, configuring a positive feedback loop that promotes HCC progression [68]. Consistently, RAGE knockdown inhibits cellular growth in HCC cell lines, while High Mobility Group Box 1 (HMGB1) stimulation increases cellular proliferation [67].

RAGE overexpression has significant functional consequences, significantly contributing to increasing the cell proliferation rhythm as well as sorafenib resistance through altered autophagy pathways [68].

In addition, the HMGB1-RAGE axis also promotes cellular migration and invasion in HCC cell lines, making it a potential therapeutic target [69].

Besides this interesting axis, the liver appears to be also particularly sensitive to several other effects mediated by AGEs, including the induction of inflammation and oxidative stress, contributing to the worsening and progression of both MASLD and HCC [63,70].

In continuity with this, the accumulation of AGEs in the hepatic tissue has been shown to promote a marked increase in low-grade chronic inflammation, a condition long recognized as a key element in carcinogenesis processes [57]. In particular, the binding between AGEs and RAGE constitutes a crucial pathogenetic event triggering several intracellular signaling pathways, including mitogen-activated protein kinase/ERK (MAPK/ERK), c-Jun N-terminal kinase (JNK), transforming growth factor beta (TGF-β), and nuclear factor kappa B (NF-κB) signaling, ultimately promoting inflammatory response and oxidative stress [51].

In particular, the activation of NF-κB stimulates the expression of adhesion molecules, further immune cell recruitment, and the production of pro-inflammatory cytokines, including IL-6 and Tumor Necrosis Factor (TNF)-α [57,71].

Notably, the activation of RAGE induced by AGEs causes an increase in the expression of the same receptor, establishing a positive feedback loop in which the ligand-dependent RAGE stimulation amplifies and maintains its biological activity over time, justifying the chronicity of a scenario dominated by a persistent low-grade systemic inflammatory state [9,51,72,73].

Persistent activation of these inflammatory pathways promotes hepatocellular injury and metabolic stress, leading to excessive production of ROS, mitochondrial dysfunction, and progressive DNA damage in hepatocytes [72]. The resulting oxidative stress contributes to genomic instability and epigenetic alterations that favor malignant transformation. In parallel, AGE–RAGE signaling promotes activation of hepatic stellate cells (HSCs) and extracellular matrix deposition, thereby fostering fibrogenesis and progressive remodeling of the hepatic microenvironment.

Chronic inflammatory signaling further activates oncogenic pathways, including STAT3 and MAPK, which stimulate hepatocyte proliferation, inhibit apoptosis, and enhance angiogenic programs through vascular endothelial growth factor (VEGF) induction [74]. Moreover, sustained cytokine production—particularly IL-6 and TNF-α—contributes to the establishment of a pro-tumorigenic niche characterized by immune dysregulation and impaired immune surveillance [74]. Comprehensively, this evidence reveals the close interrelationship between chronic inflammation, oxidative stress, and carcinogenesis [57,75], further supporting the hypothesis that AGEs may play an active role in malignant transformation and cancer progression [57]. In line with this, cumulating clinical studies indicate that patients with T2DM have increased levels of pro-inflammatory mediators, including IL-6 and TNF-α, which have been actively implicated in the development and progression of HCC, also through mechanisms related to immune escape [9,76].

**Figure 2:**
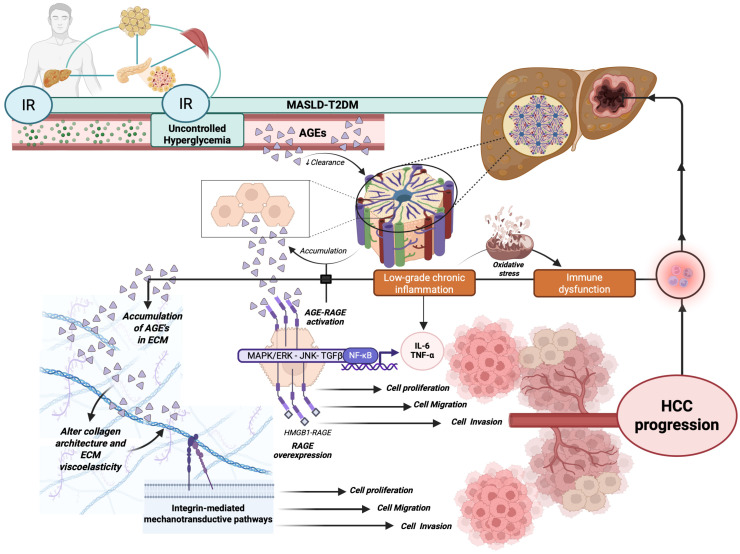
The pleiotropic effects of AGEs in the driving of hepatocancerogenesis in MASLD-T2DM. IR: Insulin resistance. AGE: Advanced Glycation End-products. RAGE: Receptor for Advanced Glycation End-products. HMGB1: High Mobility Group Box 1. ECM: Extracellular Matrix. HCC: hepatocellular carcinoma. MAPK: mitogen-activated protein kinase. ERK: Extracellular Signal-Regulated Kinase. JNK: c-Jun N-terminal kinase. NF-κB: nuclear factor kappa B. TGF-β: transforming growth factor beta. IL-6: Interleukin 6. MASLD: Metabolic dysfunction-associated steatotic liver disease. T2DM: Type 2 diabetes mellitus; arrows: induction. Created in BioRender. Romeo, M. (2026) https://BioRender.com/2cp18e5 (accessed on 14 April 2026).

Collectively, these findings also emphasize the relevance of immune dysfunction in the pathogenesis of MASLD-related liver cancer, a scenario where the interplay between chronic inflammation and IR has emerged as a crucial pathogenic axis linking diabetes, MASLD, and HCC [3]. Figure 2 summarizes the most relevant AGEs-related mechanisms contributing to hepatocancerogenesis in the setting of MASLD-T2DM (Figure 2).

### 2.3. Systemic Insulin Resistance and “Metainflammation”: The Immunometabolic Bridge Connecting MASLD, Type 2 Diabetes, and Hepatocellular Carcinoma

#### 2.3.1. Chronic “Metainflammation” Along the Adipose Tissue–Liver Axis: A Common Pathogenic Driver of Insulin Resistance in MASLD and T2DM

Chronic inflammation represents the pathogenic link connecting MD to IR, constituting a common substrate in the continuum linking T2DM, MASLD, and HCC [77]. In particular, the persistent activation of the innate immune system, driven by hepatic macrophage infiltration and the activation of pattern recognition receptors (PRRs), triggers a chronic inflammatory cascade that disrupts insulin signaling, both in hepatocytes and adipocytes [78].

Relevantly indeed, this immunometabolic dysregulation is not confined to the liver: Crosstalk between visceral adipose tissue and the liver amplifies systemic release of inflammatory cytokines and dysfunctional adipokines (leptin, resistin, adiponectin), contributing to a vicious cycle of IR and hepatic steatosis [79,80,81].

This condition, termed “metaflammation,” is characterized by persistent and systemic production of pro-inflammatory cytokines (TNF-α, IL-6, IL-1β), which aberrantly phosphorylate IRS-1 and inhibit insulin transduction through the PI3K-Akt pathway [82]. Notably, this mechanism is crucial in linking IR to gut–liver axis dysfunction, as systemic inflammation has been shown to impair intestinal barrier integrity and promote microbiota alterations [83,84,85].

Persistent activation of the innate immune system, mediated by nutrient excess, lipotoxicity, and oxidative stress [86], generates a pro-inflammatory environment that impairs insulin sensitivity [87], promotes hepatic steatosis, orchestrates inflammatory responses, and coordinates metabolic crosstalk between the liver and adipose tissue, creating a strong pathogenetic link between MASLD and T2DM [88,89]. In this context, gut-derived signals, including microbial products and metabolites, may further amplify IR-driven inflammation through activation of immune and metabolic pathways [83,84,85].

In adipose tissue, IR arises from a similar mechanism of chronic inflammation. Infiltrating M1 macrophages in adipose tissue secrete IL-1β and TNF-α, which induce serine phosphorylation of IRS-1, suppressing GLUT4-mediated glucose uptake [90]. In particular, the abundance of saturated fatty acids induces polarization of resident macrophages toward a pro-inflammatory M1 phenotype, characterized by high production of TNF-α, IL-1β, and IL-6. These cytokines interfere with insulin signaling via serine phosphorylation of IRS-1, impairing PI3K–Akt activation and reducing glucose uptake [88]. Therefore, TNF-α and IL-6 emerge as the main molecular mediators integrating inflammation and metabolism. TNF-α activates IKKβ/NF-κB and JNK pathways, promoting IRS-1 serine phosphorylation and inhibiting insulin signaling, while IL-6 activates the JAK/STAT3 pathway, increasing hepatocyte proliferation and local inflammation [90,91].

At the hepatic level, lipotoxicity resulting from the accumulation of saturated FFAs and cholesterol derivatives activates Toll-like receptors (TLR2/4) and the NF-κB pathway [90], promoting transcription of inflammatory genes and ROS production, thereby perpetuating oxidative stress and hepatic IR [92,93,94]. In vitro, exposure of primary murine hepatocytes to palmitate induces an NF-κB-dependent inflammatory response that recapitulates the alterations observed in vivo, highlighting the direct effect of saturated lipids on hepatic immunometabolic activation [95].

Studies in ob/ob and db/db mouse models have shown that genetic deletion of TLR4 significantly reduces IR and hepatic steatosis, underscoring the causal role of inflammation in modulating hepatic metabolism [88]. In addition, in vitro studies on 3T3-L1 and HepG2 cells have shown that exposure to inflammatory cytokines reduces insulin receptor tyrosine kinase activity, replicating molecular features of the insulin-resistant phenotype. Similarly, in murine MASLD models fed a high-fat diet, neutralization of IL-1β or IL-6 improves insulin sensitivity and attenuates hepatic fibrosis [96].

As previously mentioned, MASLD is strongly associated with metabolic syndrome, which includes obesity, T2DM, dyslipidemia, and hypertension [97,98]. As the global prevalence of obesity and T2DM rises, so does the prevalence of MASLD, emphasizing its connection to MD. Obesity activates various pro-inflammatory pathways, including elevated levels of cytokines such as tumor TNF-α, IL-6, CRP, plasminogen activator inhibitor-1 (PAI-1), and leptin [99,100]. These molecules are major contributors to the pathophysiology of IR. In support of this, the inhibition of JNK1 and IKK-β (which activates NF-κB) in mouse models has shown improvements in IR both locally in the liver and systemically, highlighting the role of inflammation in exacerbating IR [101]. The resulting vicious cycle between inflammation and IR fuels both conditions, acting as a continuous “file rouge” in MASLD and T2DM [16].

The pathogenic link between MASLD and T2DM is further supported by a complex network of inflammatory mediators [96]. Among these, leptin and adiponectin constitute the main regulatory duo.

Under physiological conditions, leptin enhances insulin sensitivity and glucose metabolism; however, in obesity and MASLD, leptin resistance develops, leading to hyperleptinemia, activation of inflammatory pathways such as JAK2/STAT3, NF-κB, and MAPK, and recruitment of pro-inflammatory M1 macrophages [102,103,104].

Conversely, adiponectin exerts anti-inflammatory and insulin-sensitizing effects, promoting fatty acid oxidation and inhibiting NF-κB activation [105]. Low circulating levels of adiponectin, typical in MASLD and T2DM patients, exacerbate insulin resistance and hepatic oxidative stress, favoring progression toward steatohepatitis (MASH) [106,107].

Moreover, the intrinsic relationship between immunology and metabolism can perpetuate this process and confirms the close link between MASLD and T2DM [108,109]. The immune cells in adipose tissue and the liver regulate essential metabolic functions such as lipolysis and insulin action [110,111].

These processes sustain hepatic inflammation, oxidative stress, and extracellular matrix deposition, thereby promoting the progression from simple steatosis (SS) to MASH, fibrosis, and ultimately HCC [3].

#### 2.3.2. Immunometabolic Dysregulation in MASLD-T2DM-Related Hepatocarcinogenesis

The chronically inflamed hepatic microenvironment represents only the tip of the iceberg in a pathophysiologically complex model such as MASLD, where immune dysregulation is a central determinant in disease progression up to carcinogenesis [4].

In this sense, the liver can be considered an “immunological organ” [112], and in recent years, scientific research has focused on investigating the mechanisms regulating the immune response, giving rise to the increasingly relevant concept of “trained immunity” [113].

The concept of trained immunity is linked to the existence of a form of memory in innate immune cells, with significant metabolic repercussions, or “immunometabolism” [3], emerging as a revolutionary pathogenetic model [3].

According to this theory, when stimulated and restimulated by both unspecific exogenous antigenic stimuli (β-glucan, Lipopolysaccharide LPS, or Bacillus Calmette-Guérin, BCG) and endogenous stimuli (oxidized lipids, uric acid, and heme groups) [113,114], a functional reprogramming of innate immune cells, including bioenergetic, genetic, and epigenetic changes, occurs, ultimately contributing to a shift into a pro-inflammatory phenotype [3]. In light of this, in the specific context of MASLD, chronic exposure to metabolic stressors and lipotoxic intermediates, including Damage-Associated Molecular Patterns (DAMPs) and pathogen-associated molecular patterns (PAMPs), may be deleterious [3,115], training a phenotype of hepatic innate immune cells, including Kupffer cells [116,117], HSCs, and dendritic cells (DCs) [118,119], which exhibit a pro-inflammatory profile characterized by increased production of cytokines, chemokines, and matrix-remodeling enzymes. In particular, Kupffer cells, together with monocyte-derived macrophages, represent a key node in the transition from steatosis to MASH [120]. This process is triggered by Toll-like receptor 9 (TLR-9) and Stimulator of Interferon Genes (STING), culminating in MAPK pathway activation and release of TNF-α, IL-1β, and IL-6, which interfere with IRS-1 phosphorylation, impairing insulin signaling [16,121,122]. Concurrently, activation of the NOD-Like Receptor family Pyrin domain containing 3 (NLRP3) inflammasome in hepatic cells amplifies oxidative damage, contributing to HSCs activation, inflammation, and fibrogenesis [123,124].

In progressive MASLD, the innate immune response transitions into a chronic phase that involves adaptive immune components.

CD4^+^ Th1 and Th17 cells, activated by antigen-presenting DCs and inflammatory cytokines, release interferon-gamma and IL-17A, thereby promoting hepatocyte apoptosis and HSC activation [125]. In parallel, CD8^+^ cytotoxic T lymphocytes further aggravate tissue injury through perforin and granzyme release, contributing to parenchymal damage and regenerative stress [126].

Chronic inflammation ultimately serves as a pathogenic bridge toward hepatic carcinogenesis, where persistent activation of innate immune cells, Kupffer cells, monocyte-derived macrophages, and neutrophils creates a pro-tumorigenic environment characterized by oxidative stress, compensatory proliferation, and altered immune responses [127,128,129].

Regarding this, increasing evidence indicates that MASLD-related HCC is characterized by a distinct tumor immune microenvironment (TIME), which may critically influence response to immune checkpoint inhibitors (ICIs) [130]. In this setting, chronic metabolic inflammation and persistent antigen exposure drive a progressive remodeling of the hepatic immune landscape. While early disease stages are marked by immune activation, advanced MASLD and MASLD-related HCC are typically associated with an “immune-exhausted” and immunosuppressive phenotype [130,131]. This includes the expansion of dysfunctional CD8^+^ T cells expressing inhibitory receptors such as programmed cell death protein-1 (PD-1), T cell immunoglobulin and mucin domain-containing protein-3 (TIM-3), and lymphocyte activation gene-3 (LAG-3), along with increased infiltration of regulatory T cells (Tregs) and immunosuppressive macrophages [132]. Notably, recent evidence has identified a peculiar “immune-low” phenotype in MASLD-related HCC, characterized by reduced T cell infiltration, impaired interferon signaling, and limited cytotoxic activity [132]. This immune contexture appears to be driven by lipid accumulation, oxidative stress, and metabolic reprogramming of immune cells, which collectively contribute to T cell dysfunction and impaired antitumor immunity [4].

From a translational perspective, these features may have relevant implications for immunotherapy. Accumulating clinical and preclinical evidence suggests that MASLD-related HCC may exhibit a reduced responsiveness to ICIs compared with viral-related HCC, potentially due to the predominance of immune exhaustion, altered antigen presentation, and the expansion of immunosuppressive cell populations [130,131]. In particular, metabolically driven T cell dysfunction and the enrichment of exhausted CD8^+^PD-1^+^ T cells with limited effector function may contribute to suboptimal responses to checkpoint blockade.

Altogether, these findings support the concept that MASLD-related HCC represents a unique immunological entity, in which metabolic and immune alterations converge to shape tumor behavior and therapeutic response. Through sustained immunometabolic dysregulation, inflammatory signaling not only drives systemic IR but also promotes hepatic fibrogenesis, cellular stress, and tumor-promoting remodeling of the hepatic immune microenvironment (Figure 1).

Increasing evidence suggests that these processes are further amplified by inter-organ communication networks, among which the gut–liver axis has emerged as a critical upstream regulator of metabolic and immune homeostasis (Figure 1).

In this scenario, gut–liver axis dysregulation further modulates the TIME by influencing systemic and intrahepatic immune responses, ultimately impacting immunotherapy outcomes. In particular, microbiota-derived signals have been shown to regulate immune checkpoint expression, T cell priming, and macrophage polarization, suggesting that the gut microbiome may represent a key determinant of response to ICIs in MASLD-related HCC. Therefore, a deeper understanding of this immunometabolic TIME may help identify predictive biomarkers and guide personalized immunotherapeutic strategies.

## 3. Gut–Liver Axis: The Immunometabolic Hub in MASLD-T2DM HCC

Advances in research are increasingly clarifying the role of the gut–liver axis in liver pathophysiology, consolidating its position as a key area for understanding disease mechanisms and identifying new therapeutic targets [133,134]. The gut–liver axis is a concept describing the complex bidirectional interaction between the gut (including its microbiota) and the liver [135]. This interaction is primarily established through the intestinal barrier, allowing substances present in the gut (such as nutrients and microbial-derived products) to be transported directly to the liver via the portal vein, while the liver regulates the gut microbiota by secreting molecules forming a feedback cycle whose homeostasis is maintained by the precise cooperation of specific components [136].

In pathological conditions, disruption of the intestinal barrier (“leaky gut”) permits the translocation of luminal components into the portal—and subsequently systemic—circulation due to increased intestinal permeability, representing a common pathogenic mechanism underlying various human disorders, particularly chronic liver diseases [1,18]. In this scenario, the transition from IR-driven MD to gut–liver axis dysregulation represents a key step in the elucidation of MASLD-related hepatocarcinogenesis. On one hand, IR promotes hepatic lipid accumulation and systemic inflammation, which in turn alters gut barrier integrity (“leaky gut”) and microbiota composition [84,85]. Conversely, dysbiosis-derived endotoxemia and microbial metabolites further exacerbate IR by activating inflammatory and immune pathways [23,137]. This reciprocal interaction establishes a pathogenic loop linking metabolic and immune dysfunction, thereby providing a mechanistic framework for the subsequent involvement of the gut–liver axis in promoting HCC in MASLD.

### 3.1. Dysbiosis-Induced “Leaky Gut”: Breaking the First Line of Defense in Gut–Liver Axis Homeostasis as Primum Movens in MASLD-T2DM-Related HCC

In preserving gut–liver axis homeostasis, the intestinal barrier plays a pivotal role, acting as a functional gatekeeper, defending against harmful substances through a multi-layered structure composed of a physical, chemical barrier, immune, and the gut vascular barrier (GVB) [1].

In particular, the intestinal epithelium consists of a single layer of columnar epithelial cells connected by tight junctions, which belong to the zonula occludens (ZO) families (ZO-1, ZO-2, and ZO-3) and claudins, representing a largely investigated target with several translational implications [1].

As highlighted by several recent studies, damages to the intestinal barrier are promoted by gut microbiota dysbiosis, and alterations in microbiota composition act in concert to induce intestinal barrier dysfunction, establishing a crucial bidirectional pathogenetic binomium contributing to the progression of metabolic liver disease and even hepatocarcinogenesis [138,139].

Overall, compared to healthy individuals, MD patients show higher rates of small intestinal bacterial overgrowth and gut permeability, primarily manifesting as tight junction protein disruption, as well as hepatic steatosis severity correlates with gut permeability and bacterial overgrowth [140].

In effect, specific alterations in the gut microbiota of patients with MD—particularly those affecting the gut–liver axis—constitute signatures that have been largely shown to play a significant role in the pathogenesis and progression of MASLD and T2DM [19,141,142].

Increased microbial richness has been associated with a protective effect against metabolic disorders, including obesity, metabolic syndrome, and T2DM [143]. In contrast, patients with T2DM typically exhibit reduced microbial diversity and lower metagenomic richness, conditions linked to increased IR and chronic low-grade inflammation. In addition to quantitative changes, these patients also display qualitative alterations in gut microbiota composition. Notably, one of the earliest studies investigating gut microbiota in T2DM, conducted by Larsen et al., reported reduced levels of the Firmicutes phylum and the Clostridia class [144]. The dysbiotic profile observed in T2DM is characterized by an overrepresentation of pathogenic and opportunistic Gram-negative bacteria at the expense of commensal species. These include members of the Enterobacteriaceae family, various Clostridiales, Escherichia coli, Bacteroides caccae, Lactobacilli, Prevotella copri, and Bacteroides vulgatus [145]. The enrichment of these microbial taxa is associated with increased lipopolysaccharide (LPS) levels, which promote systemic inflammation and contribute to both IR and T2DM development [146].

In this scenario, indeed, dysbiosis leads to metabolite production that can disrupt the intestinal barrier, ultimately causing bacterial and/or metabolite translocation to the liver and triggering persistent inflammation [147]. In support of this, a recent study demonstrated that fecal microbiota transplantation from HCC patients into wild-type mice caused deterioration of the intestinal barrier and translocation of viable bacteria into the liver, where they spontaneously induced inflammation, fibrosis, and dysplasia, accelerating disease progression in a murine HCC model [148].

Metagenomic analysis and bacterial culture of hepatic tissue revealed enrichment of the enteric pathogen *Klebsiella pneumoniae* in both HCC patients and mice transplanted with HCC microbiota [148]. Interestingly, the *Klebsiella pneumoniae* surface protein PBP1B interacts with and activates TLR4 on HCC cells, leading to increased cellular proliferation and activation of oncogenic signaling [148]. In line with this evidence, patients with MASLD exhibit gut dysbiosis, recurrently characterized by an increase in pathogenic bacteria such as *Escherichia coli*, *Veillonellaceae,* and particularly *Klebsiella pneumoniae* [149,150].

Dynamically, the composition of the gut microbiota can evolve, as it can be influenced by several exogenous factors, including, among others, antibiotic use and diet regimen. In humans, a Western diet (high in fat, cholesterol, and refined carbs) induces microbiome changes and intestinal dysbiosis, reducing commensals that maintain barrier integrity and increasing pro-inflammatory [151,152]. At the same time, clinical evidence reveals how obesity and high-calorie, high-fat, high-carb diets lead to dysbiosis, intestinal injury, and metabolic disorders [151,152]. In support of this, cumulative animal evidence suggests that a high-fat diet (HFD) primarily disrupts the GVB and other physical barriers, aggravating MASLD progression [153]. In HFD-induced MASLD models, C57BL/6J mice fed HFD for only one week exhibited diet-induced dysbiosis, resulting in GVB damage and bacterial translocation to the liver. This study revealed that intestinal epithelial barrier and GVB disruption are early events in MASH pathogenesis and that inhibiting GVB disruption prevents MASH development [153].

In MASLD/MASH, few studies have properly explored the specific mechanisms by which bacterial metabolites influence the intestinal barrier. High intestinal levels of ethanolamine in obese mice upregulate microRNA-101a-3p expression, reducing tight junction protein mRNA stability and ultimately weakening barrier function [83]. On the other hand, the supplementation with a bioactive dietary fiber (glucomannan) promoted the growth of *Bacteroides ovatus* in HFD-fed mice and, relevantly, the indole-3-acetic acid produced by *B. ovatus* is a key bioactive metabolite that enhances barrier function via aryl hydrocarbon receptor activation, improving IR [154].

Additionally, *Parabacteroides distasonis* and its pentadecanoic acid metabolite have been shown to improve MASH by restoring barrier function and preventing bacterial toxin translocation [155].

Altogether, this evidence supports the role of the intestinal barrier as the last line of defense safeguarding gut–liver axis homeostasis. When its structural and functional integrity is compromised, the intestinal epithelium loses its capacity to effectively contain luminal microbial components, allowing the translocation of bacteria and microbiota-derived products into the portal circulation. As a consequence, the liver—continuously exposed to gut-derived signals—becomes a primary target of these inflammatory stimuli. The resulting exposure to pathogen-associated molecular patterns and microbial metabolites triggers the activation of hepatic innate immune pathways, ultimately promoting a persistent inflammatory state that contributes to MD and progressive liver injury.

However, emerging evidence suggests that this portal macrophage population may function as an additional defensive checkpoint along the gut–liver axis, representing a second line of immune surveillance that filters gut-derived inflammatory signals before they reach the hepatic parenchyma. Failure of this regulatory system may therefore facilitate the propagation of inflammatory stimuli to the liver, promoting the development of inflammatory liver diseases such as MASH. In support of this, a recent study revealed that the portal area is enriched with immunosuppressive macrophages expressing high levels of IL-10 and the “Macrophage receptor with collagenous structure” (MARCO), a class A scavenger receptor capable of sequestering pro-inflammatory PAMPs and DAMPs, thereby inhibiting immune responses [156]. Interestingly, the induction of MARCO^+^ immunosuppressive macrophages is regulated by the gut microbiota, with commensal bacteria limiting excessive hepatic inflammation, remarking on the concept that the gut microbiota acts as a key signaling generator, interacting with the host through its metabolites.

### 3.2. Microbiota-Derived Metabolites Regulate Inflammation and Immune Signaling in Hepatocancerogenesis

#### 3.2.1. Breaking Intestinal Barriers: Metabolic Endotoxemia and Systemic Inflammation

Accumulating evidence indicates that gut microbiota alterations contribute significantly to HCC development and progression by modulating multiple pathways along the gut–liver axis.

In this context, it has been comprehensively demonstrated that the intestinal flora of HCC patients exhibits elevated levels of Gram-negative bacteria. This association is closely linked to increased serum levels of LPS and endotoxemia, leading to chronic systemic inflammation [157].

As previously shown, in gut–liver axis dysregulation, increased intestinal permeability facilitates the translocation of microbial products, especially LPS, into portal circulation. These components activate liver inflammatory responses via TLR, promoting chronic inflammation, fibrogenesis, and hepatocyte neoplastic transformation [158]. In this sense, LPS represents the main microbe-associated molecular pattern (MAMP) recognized by receptors on innate immune cells, including TLRs.

When activated by pathogens or commensals, TLRs trigger immune responses via NF-κB induction, ultimately enhancing the production of inflammatory cytokines [159,160].

In support of this, Lin et al. reported that TLR4-deficient mice exhibited reduced liver injury and inflammatory responses compared to wild-type mice [161], and Dapito et al. found a higher expression of TLR4 in HCC tissues (77.8%) compared with adjacent nontumor tissues [162].

Among species involved in inflammatory-mediated HCC pathogenesis, emerging data suggest *Escherichia coli* and *Bacteroides fragilis*, an opportunistic pathogen in GI tumors, as significant contributors to hepatocarcinogenesis through TLR4-dependent immune modulation [163]. In particular, *Escherichia coli*, frequently enriched in MASLD-T2DM-related dysbiosis, by producing high LPS levels activating TLR4 on Kupffer cells and hepatocytes, determines chronic liver inflammation and oxidative stress that crucially drives hepatocarcinogenesis [164]. Conversely, *Bacteroides fragilis* has been shown to promote tumor-supporting inflammation through the release of virulence factors, which can induce a senescence-associated secretory phenotype in HSCs, thereby fostering a pro-inflammatory and pro-tumorigenic hepatic microenvironment [165].

In this scenario, recent evidence highlights epiregulin as a pivotal stromal mediator linking gut-derived endotoxemia to hepatocarcinogenesis. Using a xenograft model combining EGFR^+^ HCC cells with HSCs, Kubo et al. showed that LPS markedly enhances tumor growth by inducing epiregulin in the tumor stroma, activating paracrine EGFR signaling in cancer cells [166]. This axis promotes tumor proliferation, invasiveness, and pro-angiogenic programs via IL-8 upregulation and increased CD34^+^ neovascularization, supporting stromal-targeted and microbiota-oriented HCC strategies [166].

Additionally, and in continuity with this, gut flora with relative metabolites have also been shown to influence tumor immune responses in HCC patients, by modulating the functioning of several immune cell phenotypes, including macrophages [167], Tregs [168], B cells [169], CD8+ T cells [170], and CD4+ T cells [171]. In particular, gut-derived signals have been shown to modulate macrophage polarization, favoring either pro-inflammatory or immunosuppressive phenotypes depending on the microbial context. For instance, dysbiosis-associated LPS can activate Toll-like receptor signaling pathways in hepatic macrophages, promoting the production of inflammatory cytokines that contribute to chronic liver inflammation and tumor progression [168]. At the same time, microbial metabolites may also influence the recruitment and functional programming of regulatory T cells (Tregs) [172], which play a crucial role in maintaining immune tolerance but, in the tumor context, can suppress effective antitumor immunity.

Beyond macrophages and Tregs, the gut microbiota has also been implicated in modulating B cell responses and the activity of cytotoxic and helper T lymphocytes [173]. Alterations in microbiota composition may impair the cytotoxic activity of CD8^+^ T cells, which are essential for tumor surveillance and elimination, while also affecting the differentiation of CD4^+^ T helper subsets [174]. These changes can lead to a weakened antitumor immune response and contribute to the establishment of an immunosuppressive tumor microenvironment.

Collectively, these observations highlight the pivotal role of the gut microbiota as a regulator of tumor immunity in HCC, suggesting that microbiota-driven immune modulation may represent an important mechanism linking gut–liver axis dysfunction to hepatocarcinogenesis. In this sense, these findings emphasize the gut microbiome’s critical role in shaping the hepatic immune microenvironment and driving oncogenesis. Targeted modulation of the gut microbiota may represent a novel adjunct for HCC prevention and therapy. Importantly, the impact of the gut microbiota on hepatocarcinogenesis extends beyond microbial components to include a wide range of microbiota-derived metabolites, among which bile acids, tryptophan metabolites, and short-chain fatty acids represent key signaling molecules shaping intestinal permeability, as well as metabolic and immune pathways along the gut–liver axis.

#### 3.2.2. Bile Acid Signaling and Immune–Metabolic Crosstalk

Beyond PAMPs/MAMPs-mediated TLR activation, dysregulated bile acid metabolism is another key microbiota-related mechanism in hepatocarcinogenesis [175]. Primary bile acids (cholic acid and chenodeoxycholic acid) are synthesized in the liver from cholesterol and conjugated with glycine or taurine before secretion into the bile. These primary bile acids are only minimally metabolized by the host and reach the colon, where diverse bacterial communities metabolically transform them into secondary bile acids through 7-alpha-dehydroxylation reactions catalyzed by bacterial bile salt hydrolases (BSHs) and specialized metabolic enzymes [176].

The dysbiotic microbiota in MASLD-T2DM exhibit substantially altered bile acid metabolic capacity.

The loss of strict anaerobes and specialized bile acid degraders such as Clostridium clusters IV and XIVa results in diminished conversion of primary to secondary bile acids, altering the ratio of primary to secondary bile acids in the colon and portal circulation [175].

Additionally, dysbiotic microbiota may show altered production of specific secondary bile acid species: deoxycholic acid (DCA), generated through 7-alpha-dehydroxylation of cholic acid, is produced at higher levels by some dysbiotic communities, while ursodeoxycholic acid (UDCA), which exhibits more immunoprotective properties, is produced at lower levels [175]. This alteration of secondary bile acid composition has profound downstream consequences, as different bile acid species possess distinct affinities for farnesoid X receptor (FXR) and Takeda G-protein coupled receptor 5 (TGR5), the primary targets through which they exert crucial immunological effects [177].

FXR, a nuclear receptor, is activated by both primary and secondary bile acids, with secondary bile acids (particularly DCA and lithocholic acid—LCA) showing higher affinity for FXR than primary bile acids. Upon activation, FXR translocates to the nucleus and acts as a transcription factor, regulating genes involved in bile acid synthesis, cholesterol metabolism, and inflammatory responses. FXR activation in macrophages and intestinal epithelial cells suppresses NF-κB-mediated transcription of pro-inflammatory cytokines, including TNF-α and IL-6, thereby reducing inflammation [178].

TGR5, by contrast, is a G-protein-coupled receptor located on the cell surfaces of macrophages, dendritic cells, and intestinal epithelial cells. All bile acids activate TGR5, but secondary bile acids typically show higher potency than primary bile acids. TGR5 activation triggers rapid intracellular signaling through Gαs-coupled cAMP production and activation of downstream PKA and AMPK pathways [179]. In macrophages, TGR5 activation leads to reduced production of pro-inflammatory cytokines through mechanisms involving cAMP-mediated suppression of NF-κB translocation. Additionally, TGR5 activation in intestinal epithelial cells enhances epithelial barrier function and promotes mucus production, while in enteroendocrine cells, TGR5 activation stimulates GLP-1 secretion, linking bile acid signaling to glucose homeostasis.

In MASLD-T2DM, the dysbiotic alterations in secondary bile acid metabolism result in dramatic changes in the composition and concentration of bile acid species reaching the liver and peripheral circulation [175]. Gut microbes modify bile acid composition and hydrophobicity through enzymatic activities, notably bile salt hydrolase (BSH), deconjugating primary BAs and increasing free, hydrophobic BAs such as deoxycholic acid (DCA) and lithocholic acid (LCA). These cytotoxic BAs induce ER stress, mitochondrial dysfunction, oxidative damage, and hepatocyte death [165]. While absolute fecal bile acid concentrations may be maintained, the relative proportions of individual bile acid species shift toward primary bile acids and potentially pathogenic secondary bile acids at the expense of more immunoprotective species. This compositional shift reduces the overall signaling through both FXR and TGR5, diminishing the anti-inflammatory and tolerance-promoting effects of bile acid signaling [165].

The portal circulation in MASLD-T2DM consequently delivers an altered bile acid signal to the liver. At the hepatic level, bile acids selectively modulate hepatic immune and nonimmune cells, including macrophages, T cells, HSCs, and LSECs. Chronic accumulation of toxic BAs fosters a pro-inflammatory, carcinogenic hepatic microenvironment [180], contributing to hepatocyte transformation and malignant progression.

The diminished secondary bile acid availability impairs FXR and TGR5 signaling in hepatic macrophages (Kupffer cells), reducing their production of IL-10 and TGF-β while potentially permitting increased NF-κB-driven pro-inflammatory cytokine production in response to concurrent LPS signaling [181]. Similarly, altered bile acid composition affects intestinal FXR and TGR5 signaling, reducing epithelial barrier strengthening and immune tolerance signals at a time when dysbiosis has compromised barrier integrity and activated pro-inflammatory innate immunity.

Beyond their metabolic and inflammatory roles, bile acids have recently emerged as critical regulators of antitumor immunity and response to ICIs [182]. Increasing evidence indicates that alterations in bile acid composition, particularly in dysbiotic conditions such as MASLD, can profoundly influence T cell functionality and contribute to immune suppression within the tumor microenvironment [183]. In particular, specific bile acid species have been shown to impair CD8^+^ T cell activation and promote T cell exhaustion through multiple mechanisms, including modulation of antigen-presenting cell function and induction of immunosuppressive signaling pathways [184]. Mechanistically, secondary bile acids can influence the recruitment and expansion of regulatory T cells and myeloid-derived suppressor cells, and the bile acid-driven metabolic reprogramming of immune cells has been associated with reduced cytotoxic T cell activity and impaired antitumor responses, ultimately reinforcing an immunosuppressive milieu [185]. In support of this, sirtuin 5 (SIRT5) loss has been demonstrated to promote bile acids-driven liver tumorigenesis by creating an immunosuppressive TME. In general, SIRT5 deficiency dysregulates bile acid homeostasis, leading to the accumulation of tumor-promoting bile acids, enhancing myeloid-derived suppressor cell infiltration, and suppressing CD8^+^ T cell function, facilitating immune escape [186]. Consistent with this, reduced SIRT5 expression in human HCC correlates with poor clinical outcomes [186].

From a translational perspective, emerging studies suggest that specific bile acid profiles may correlate with responsiveness to immunotherapy, supporting their potential role as predictive biomarkers. Relevantly, therapeutic modulation of bile acid signaling pathways may represent a novel strategy to restore immune competence and enhance the efficacy of ICIs in MASLD-related HCC [185].

#### 3.2.3. Tryptophan Metabolites and Aryl Hydrocarbon Receptor (AhR) Signaling: A Master Regulator of Intestinal Barrier and Immune Functioning

Tryptophan, an essential amino acid present in dietary proteins, serves as a substrate for multiple microbial metabolic pathways in the colon, generating a diverse array of immunologically active products [187]. The bacterial tryptophanase enzyme catalyzes the deamination of tryptophan to generate indole, which is further metabolized by both bacterial and host enzymes to produce indole-3-aldehyde (I3A), indole-3-acetic acid (IAA), indole-3-propionic acid (IPA), skatole, and other indole derivatives [188]. These indole metabolites serve as ligands for the aryl hydrocarbon receptor, a ligand-activated nuclear receptor that functions as a sensor of environmental xenobiotics and dietary components but also responds to endogenous microbially produced signals.

The host also possesses enzymes capable of tryptophan catabolism, particularly the indoleamine 2,3-dioxygenase (IDO1) and tryptophan 2,3-dioxygenase (TDO2) enzymes, which initiate the kynurenine pathway in both innate immune cells and intestinal epithelial cells [189].

Through this pathway, tryptophan is converted to kynurenine, which undergoes sequential enzymatic conversions to generate downstream products, including quinolinic acid, anthranilic acid, and xanthurenic acid, many of which activate AhR signaling. The balance between microbially produced indole derivatives and host-produced kynurenine metabolites determines the overall AhR activation landscape in the intestinal mucosa and systemically.

In this scenario, the AhR represents a critical nexus between microbiota-derived signals and immune homeostasis, functioning as both a xenobiotic sensor and a pleiotropic regulator of developmental processes and age-related tissue degeneration [190]. When activated by ligands including both exogenous xenobiotics and endogenous tryptophan metabolites, AhR translocates to the nucleus, heterodimerizes with the aryl hydrocarbon nuclear translocator (ARNT), and binds to DNA sequences called dioxin response elements (DREs) to transcriptionally activate target genes. In the context of intestinal immunity, AhR activation exerts profound immunoregulatory effects.

In particular, it promotes IL-22 production by innate lymphoid cells (particularly ILC3s) and CD4+ T cells, drives IL-10 production from myeloid cells and epithelial cell-associated T cells, and suppresses Th17 differentiation through reduction of RORγt expression.

AhR is abundantly expressed in intestinal epithelial cells, intraepithelial lymphocytes, and mucosal dendritic cells, positioning it to integrate microbial signals throughout the intestinal immune system [178]. Activation of AhR in intestinal epithelial cells initiates signaling cascades that enhance tight junction integrity through upregulation of claudins, occludin, and junction adhesion molecules, directly strengthening the epithelial barrier against pathogen translocation. Additionally, AhR-mediated IL-22 production from innate lymphoid cells stimulates intestinal epithelial production of antimicrobial peptides (RegIII gamma, lipocalin-2) and mucus-associated genes (MUC2, MUC4), creating enhanced antimicrobial defenses and physical barriers against bacterial translocation.

Interleukin-22, a member of the IL-10 cytokine family, plays a dominant role in maintaining intestinal homeostasis and epithelial barrier function [187]. IL-22 is primarily produced by innate lymphoid cells (ILC3s), γδ T cells, and CD4+ Th17/Th22 cells.

Once secreted, IL-22 binds to the IL-22 receptor complex on intestinal epithelial cells, initiating STAT3 phosphorylation and activation of downstream MAPK pathways that drive epithelial cell survival, proliferation, and enhanced mucus production [187]. The IL-22-STAT3 axis promotes epithelial cell production of tight junction proteins, including claudins and occludin, which are essential components of epithelial tight junctions [187].

Furthermore, IL-22-stimulated epithelial cells upregulate genes encoding antimicrobial peptides, including the REG family of C-type lectins and lipocalin-2, which have direct bacteriostatic and bactericidal activity against Gram-negative and Gram-positive bacteria [191]. This IL-22-mediated response strengthens the intestinal epithelial barrier through multiple complementary mechanisms: enhanced tight junction protein expression reduces paracellular pathogen translocation, while increased antimicrobial peptide production reduces bacterial load and pathobiont colonization.

Dysbiosis-associated reductions in AhR ligand availability impair IL-22 production, leading to reduced epithelial tight junction protein expression and increased susceptibility to LPS and pathogen translocation.

Interestingly, multiple lines of evidence indicate that dysbiosis in MASLD-T2DM is accompanied by significant dysregulation of tryptophan metabolism, both microbial and host-derived. Dysbiotic microbiota depleted of diverse tryptophan-metabolizing bacteria show substantially reduced capacity for generating protective indole derivatives [175]. Simultaneously, dysbiotic conditions often promote expansion of bacteria that express tryptophanase variants with altered substrate specificity or reduced expression, further reducing indole production. Additionally, dysbiotic microbiota typically show increased expression of indole-degrading enzymes from pathobionts, consuming indole and limiting its availability for host AhR activation.

Host-derived tryptophan metabolism via the kynurenine pathway becomes dysregulated in MASLD-T2DM, with chronic inflammation driving elevated IDO1 and TDO expression in macrophages and dendritic cells, thereby increasing tryptophan consumption toward kynurenine production at the expense of other pathways [190]. The balance between protective and pathogenic kynurenine pathway metabolites becomes dysregulated: accumulation of kynurenine itself (which can exhibit both AhR-agonistic and immunosuppressive effects) combines with increased production of neurotoxic downstream metabolites like quinolinic acid, which may contribute to both intestinal and systemic immunosuppression. This dysregulation of tryptophan metabolism contributes to the downregulation of AhR expression and function that has been observed in dysbiotic intestinal samples, particularly in inflammatory contexts. The resulting defect in AhR signaling removes a critical molecular brake on intestinal inflammation, permitting expansion of Th17 cells, reduced IL-22 production, and progressive epithelial barrier dysfunction [190].

Interestingly, beyond microbial composition and intestinal permeability, additional mediators of gut–liver communication are increasingly being recognized [192]. Among these, the vagus nerve represents an important neuroimmune interface linking intestinal signals with hepatic metabolism and inflammation [192]. Through bidirectional signaling, vagal pathways may modulate glucose homeostasis, immune activation, and inflammatory reflex mechanisms, thereby potentially influencing MASLD progression [192,193]. In parallel, microbiota-derived metabolites such as trimethylamine N-oxide (TMAO), generated from dietary choline and carnitine, have emerged as relevant contributors to MD [193]. Elevated circulating TMAO levels have been associated with IR, hepatic steatosis, inflammation, and fibrosis, while experimental evidence suggests that TMAO may promote oxidative stress, inflammatory signaling, and a pro-carcinogenic microenvironment [193,194]. Although further studies are needed, these observations support the concept that neuroimmune pathways and non-classical microbial metabolites may represent additional components of the gut–liver axis involved in MASLD-related disease progression.

#### 3.2.4. Short-Chain Fatty Acids (SCFAs) as Key Immunoregulatory Metabolites

Short-chain fatty acids (SCFAs), primarily including acetate (2-carbon), propionate (3-carbon), and butyrate (4-carbon), are end products of anaerobic bacterial fermentation of indigestible carbohydrates and fiber [177]. These molecules serve not only as energy substrates for colonocytes but also as signaling molecules that communicate microbial metabolic status to the host immune and metabolic systems. In healthy individuals consuming adequate dietary fiber, fecal SCFA concentrations typically range from 50 to 100 mM, with butyrate comprising approximately 15–20% of total SCFAs [177]. Dysbiotic microbiota, particularly those depleted of anaerobic SCFA producers, often show markedly reduced fecal SCFA levels, though some compensation may occur through pathobiont-derived production.

The absorption and systemic distribution of SCFAs depend on colonic pH and bacterial metabolic activity. SCFAs produced in the cecum and proximal colon are rapidly absorbed through epithelial transporters, including the monocarboxylate transporters (MCTs) and the sodium-coupled SCFA transporter (SLC5A8) [195]. Absorbed SCFAs enter the portal blood and are transported directly to the liver, where they exert metabolic effects on hepatic glucose production and fatty acid synthesis. SCFAs exert many of their immunoregulatory effects through activation of cell-surface G-protein-coupled receptors, including GPR41 (FFAR3), GPR43 (FFAR2), and GPR109A (HCAR2) [177]. These receptors are expressed on multiple immune cell types, including intestinal innate lymphoid cells (ILCs), dendritic cells, T cells, and macrophages, as well as on intestinal epithelial cells.

GPR43 activation in specific immune populations engages β-arrestin/ERK or AMPK-linked pathways depending on cell context, generating cell-type-specific responses ranging from Treg induction to inhibition of pro-inflammatory cytokine production [191]. Additionally, circulating SCFA levels influence systemic immune responses: butyrate can cross the blood–brain barrier via MCTs and directly modulate neuroinflammation and microglial activation. In MASLD-T2DM-related dysbiosis, reduced bacterial fermentation combined with altered epithelial transporter expression results in diminished systemic SCFA availability, even when some SCFA production persists.

Although all three major SCFAs bind to common receptors, they demonstrate distinct immunological properties that merit detailed consideration.

Butyrate, the most thoroughly studied SCFA in immunological contexts, at physiologically plausible concentrations (10–100 μM in mucosal tissues), suppresses pro-inflammatory ILC2 proliferation and Th17 differentiation while promoting differentiation and expansion of Foxp3+ regulatory T cells (Tregs) and IL-10-producing B cells [196]. The spatial and temporal expression patterns of these receptors create tissue-specific effects of SCFAs. In purified intestinal ILC2s, ex vivo reductions in the lineage transcription factor GATA3 and IL-13/IL-5 cytokine production occur at butyrate concentrations of 10-200 μM, indicating physiologically plausible dose windows for metabolite-driven immune modulation [191].

GPR41 activation on intestinal dendritic cells promotes a tolerogenic phenotype characterized by reduced IL-12 production and increased IL-10 and TGF-β output, driving Treg differentiation in inductive sites [178]. This SCFA-receptor signaling cascade plays a critical role in maintaining intestinal immune homeostasis under normal conditions; dysbiosis-driven SCFA depletion abolishes this signal, thereby promoting default pro-inflammatory activation of immune cells. In support of this, McBrearty et al. demonstrated SCFAs’ protective role in hepatocarcinogenesis using HBx transgenic mice, showing delayed HCC onset, reduced tumor burden, attenuated hepatic inflammation, oxidative stress, hepatocyte proliferation, and modulation of NF-κB signaling [197].

Moreover, butyrate acts as an epigenetic modifier by inhibiting histone deacetylases (HDACs), thereby increasing histone acetylation at promoter regions of genes encoding anti-inflammatory mediators and tight junction proteins [191]. In this sense, indeed, the most significant immunological function of butyrate involves its capacity to inhibit HDACs, a family of enzymes that remove acetyl groups from histone lysine residues, thereby increasing chromatin compaction and transcriptional repression [178]. HDAC inhibition by butyrate elevates global histone acetylation within immune cells, creating a permissive chromatin state for transcription of anti-inflammatory genes, including IL-10, TGF-β, and tight junction proteins.

Furthermore, HDAC inhibition specifically increases acetylation of the Foxp3 locus, stabilizing the differentiation and function of Tregs even in inflammatory microenvironments. Relevantly, in MASH models, butyrate induces macrophage M2 polarization via HDAC inhibition, limiting pro-tumorigenic hepatic inflammation [198].

Anyway, the epigenetic effects of butyrate extend beyond chromatin remodeling to include direct acetylation of non-histone proteins, including transcription factors, signaling molecules, and metabolic enzymes [199]. Acetylation of p65 (RelA) subunits of NF-κB by acetyl-CoA-dependent acetyltransferases following HDAC inhibition increases p65 binding to coactivators while decreasing its binding to corepressors, paradoxically enhancing NF-κB-driven transcription in some contexts [199]. However, HDAC inhibition also acetylates histone H3 and H4 at the promoters of pro-inflammatory cytokine genes, generally suppressing their expression in myeloid cells. Thus, the net effect of butyrate’s epigenetic actions is typically immunosuppressive in the setting of active TLR signaling [199].

Propionate and acetate, while sharing some metabolic functions with butyrate, possess distinct immunological profiles.

Propionate exhibits somewhat lower HDAC inhibitory activity than butyrate but efficiently activates GPR43 (FFAR2), triggering β-arrestin and AMPK-dependent signaling in specific immune cell populations [191]. This G-protein-coupled receptor signaling can activate multiple parallel immune regulatory pathways, including AMPK-dependent suppression of mTOR signaling and consequent Treg expansion.

Acetate, the most abundant SCFA in the colon, primarily acts through GPR43 and GPR41 signaling and additionally serves as a substrate for histone acetylation through acetyltransferase activity, contributing to epigenetic reprogramming. The loss of all three SCFA producers in dysbiosis therefore eliminates multiple, partially redundant immunoprotective signals. Relevantly, Hu et al. showed microbiota-derived acetate suppresses IL-17A by modulating ILC3 function via HDAC inhibition, enhancing PD-1/PD-L1 blockade efficacy [200].

### 3.3. Gut Metabolite-Driven Immune Dysregulation: A Network Promoting Hepatocarcinogenesis

The progression from dysbiosis to hepatic inflammation to HCC involves sustained, dysbiosis-driven activation of NF-κB signaling in hepatocytes, Kupffer cells, and HSCs [176]. As previously shown, this progression occurs through multiple parallel and converging pathways: metabolic endotoxemia activates TLR4 on Kupffer cells and hepatocytes, leading to MyD88-dependent phosphorylation and degradation of IκBα with consequent nuclear translocation of p65/p50 NF-κB dimers [176]. NF-κB-driven transcription in response to sustained TLR4 activation initiates production of multiple pro-carcinogenic mediators, TNF-α, IL-6, IL-1β, and other pro-inflammatory cytokines, that perpetuate hepatic inflammation and promote hepatocellular apoptosis and regeneration [201]. The hepatic stellate cell, normally quiescent and maintaining the extracellular matrix, becomes activated toward a pro-fibrotic phenotype in response to TNF-α and TGF-β signals from inflammatory macrophages and damaged hepatocytes.

Activated HSCs produce excessive collagen and other matrix proteins, initiating hepatic fibrosis [201]. Furthermore, TNF-α and IL-6 promote the proliferation of surviving hepatocytes through paracrine signaling, creating a permissive environment for clonal expansion of cells with oncogenic mutations. The combined effects of sustained inflammation, hepatocellular regeneration, and accumulating genomic damage drive progression toward HCC [200].

Simultaneously, reduced SCFA availability eliminates the HDAC-inhibitory signals that normally restrain NF-κB-driven transcription. Additionally, reduced secondary BA signaling removes FXR-mediated suppression of NF-κB [176]. The convergence of multiple pro-inflammatory signals on a background of diminished counter-regulatory metabolite signals creates a permissive environment for constitutive NF-κB activation.

Besides this, a critical mechanism by which dysbiosis-driven immune dysfunction contributes to HCC development involves dysregulation of immune checkpoints and loss of antitumor immunity [199]. Dysbiotic microbiota show dramatically reduced capacity for SCFA production and altered tryptophan metabolism, eliminating signals that normally drive differentiation and maintenance of functional tumor-fighting CD8+ T cells and antitumor Th1/Th17 responses.

The combination of sustained chronic inflammation with reduced numbers of functional effector T cells creates a permissive microenvironment for malignant cell growth.

The specific metabolite-driven effects on immune checkpoints involve both direct modulation of checkpoint ligand expression and regulation of the T cell exhaustion program. Dysbiosis-derived accumulation of pro-inflammatory metabolites, including succinate and LPS, promotes sustained expression of PD-L1 on tumor-infiltrating macrophages, myeloid-derived suppressor cells, and eventually on HCC cells themselves [199].

Mechanistically, succinate-driven metabolic reprogramming in myeloid cells enhances stabilization of hypoxia-inducible factor 1-alpha (HIF-1α), which transcriptionally activates PD-L1. Additionally, dysbiosis-associated reduced indole derivative availability impairs AhR-mediated IL-22 production, removing a critical signal that maintains CD8+ T cell function and prevents T cell exhaustion [199].

In this framework, beyond its role in driving hepatocarcinogenesis, dysbiosis and gut-derived signals emerge as factors that critically influence the response to ICIs [202,203]. In line with this, in the MASLD–T2DM context, gut microbiota composition undergoes profound alterations, and accumulating evidence indicates that these changes are closely associated with differential responses to ICIs, with distinct microbial signatures and levels of bacterial diversity observed between responders and non-responders. Dysbiotic profiles characterized by enrichment of pro-inflammatory taxa and depletion of beneficial commensals—particularly butyrate-producing bacteria and Akkermansia muciniphila—have been associated with impaired immunotherapy efficacy, whereas enrichment of taxa such as Lachnospiraceae and related genera correlates with improved clinical outcomes [204].

In particular, Akkermansia muciniphila has emerged as a key modulator of ICI responsiveness, through restoration of intestinal barrier integrity, reduction of endotoxemia, and modulation of immunosuppressive cell populations such as myeloid-derived suppressor cells and M2 macrophages [204]. Conversely, pro-inflammatory metabolites, including LPS and trimethylamine N-oxide, further amplify NF-κB-driven inflammation and reinforce resistance to immunotherapy [203].

Importantly, external factors capable of altering gut microbiota composition may significantly impact therapeutic outcomes. In particular, antibiotic exposure has been associated with impaired responses to ICIs, likely due to disruption of beneficial microbial communities involved in immune activation [205,206]. Conversely, strategies aimed at restoring microbial balance—including dietary interventions, probiotics, prebiotics, or fecal microbiota transplantation—are currently being explored as potential approaches to enhance immunotherapy responsiveness [205,207].

Overall, these observations highlight gut microbiota and intestinal circulation as dynamic regulators of both hepatocarcinogenesis and immunotherapy response in MASLD-related HCC [205].

**Figure 3:**
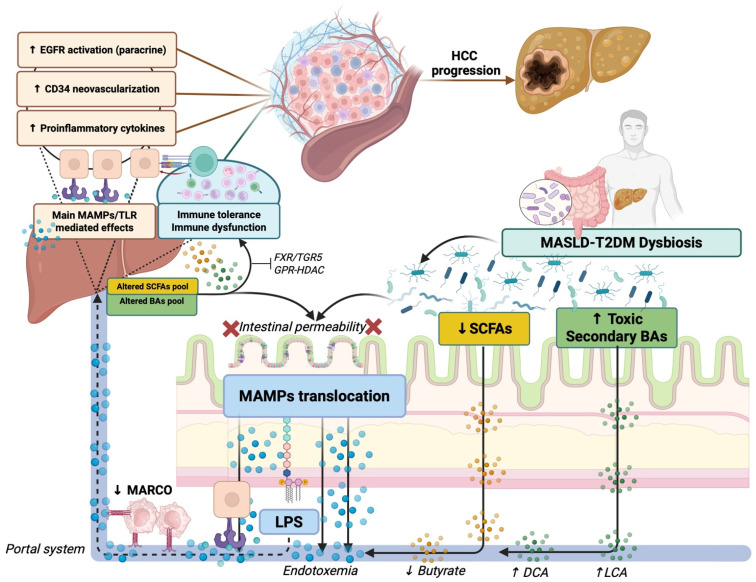
Gut-derived metabolites influence immunometabolic pathways driving HCC in MASLD-T2DM. HCC: Hepatocellular carcinoma. MASLD: Metabolic dysfunction-associated steatotic liver disease. T2DM: Type 2 diabetes mellitus. SCFAs: short-chain fatty acids. BAs: Bile acids. MAMPs: microbe-associated molecular patterns. LPS: Lipopolysaccharide. FXR: Farnesoid X receptor. TGR5: Takeda G-protein coupled receptor 5. HDAC: histone deacetylases. MARCO: Macrophage receptor with collagenous structure. TLR: Toll-like receptor. EGFR: epidermal growth factor receptor; Arrow: induction/passage; ↑: increase, ↓: decrease. Created in BioRender. Romeo, M. (2026) https://BioRender.com/2cp18e5 (accessed on 14 April 2026).

Figure 3 summarizes the impact of gut-derived metabolites on immunometabolic pathways significantly involved in creating a microenvironment where hepatocancerogenesis is promoted in the setting of MASLD-T2DM (Figure 3).

Comprehensively, the emerging understanding of how microbiota-derived metabolites regulate hepatocarcinogenesis has revealed a nuanced, context-dependent framework [208]. Rather than exhibiting fixed pro- or antitumorigenic activities, metabolites such as secondary bile acids, SCFAs, and indole derivatives demonstrate bidirectional effects that depend on their concentration, the specific host immune status, tumor microenvironment characteristics, and the activation patterns of their cognate receptors.

This context-dependency framework explains apparent paradoxes in the literature and provides a mechanistic basis for understanding how metabolite dysfunction contributes to HCC development in MASLD-T2DM [208].

Mechanistically, these metabolites operate as systemic "remote immunoregulators" that reshape tumor immunity and hepatic immunity through multiple integrated pathways [209]. They function as ligands for specific cell surface and nuclear receptors, modulating epigenetic programs, metabolic reprogramming, and T cell effector function. The same metabolite can either potentiate immune surveillance or entrench immunosuppression depending on ligand–receptor pairing, dose, and tissue niche [208]. Understanding these metabolite-immune circuits is essential for developing rational interventions that modify the microbiota to prevent or arrest hepatocarcinogenesis in MASLD-T2DM.

Altogether, this evidence depicts a complex scenario where the reciprocal interactions between intestine barrier, microbiota, and liver mediated by gut-derived metabolites (including MAMPs, BAs, SCFAs, particularly) significantly influence HCC development and progression [153,210]. Moreover, taken together, these findings also highlight the gut–liver axis as a central driver of immunometabolic dysregulation and hepatocarcinogenesis in the MASLD–T2DM setting. In this sense, the growing understanding of the molecular and cellular mechanisms linking gut microbiota alterations, microbial metabolites, and immune remodeling to liver cancer development has opened new perspectives for therapeutic intervention. In this context, targeting the gut–liver axis is increasingly emerging as a promising strategy with potential translational and therapeutic implications for the management of MASLD–T2DM-associated HCC.

## 4. From Bench to Bedside: Exploring the Translational Implications of Integrating the Targeting of the Gut–Liver Axis in the Management of Patients with MASLD-T2DM-Associated HCC

Hepatocellular carcinoma (HCC) remains one of the leading causes of cancer-related mortality worldwide, despite substantial therapeutic advances over the past decade. Current treatment strategies are based on a stage-adapted approach that includes curative therapies (surgical resection, liver transplantation, and ablation), locoregional treatments, and systemic therapies, mainly multikinase inhibitors and, more recently, combinations of immune checkpoint inhibitors and anti-angiogenic agents. However, meaningful clinical benefit is largely confined to early disease stages and is burdened by marked interindividual heterogeneity in treatment response and significant tolerability issues, particularly in patients with advanced cirrhosis [211,212,213].

In more advanced stages, immune checkpoint inhibitors have opened new and promising therapeutic avenues for HCC. Nevertheless, response rates remain variable, and a substantial proportion of patients fail to derive significant benefit. In this context, strategies aimed at modulating the gut microbiota have emerged as a potential means to enhance therapeutic efficacy in liver cancer [214,215].

### 4.1. Therapeutic Applications: Targeting IR Is a Crucial, Even Limited Part of the Puzzle

In the last decade, several animal findings have provided fundamental causal evidence linking IR, MD, T2DM, and hepatocarcinogenesis. In support of this, Jeong et al. developed an innovative male mouse model combining a hypercaloric diet, systemic metabolic dysfunction, and spontaneous progression to HCC, closely recapitulating the clinical continuum observed in patients with MASLD and T2DM [216].

In this model, mice develop visceral obesity, marked IR, hyperglycemia, and hyperinsulinemia, followed by steatohepatitis, advanced fibrosis, and HCC [216].

The relevance of hepatic metabolic flexibility has been further highlighted by the study of Gallage et al., who demonstrated that a 5:2 intermittent fasting regimen significantly reduces steatohepatitis, fibrosis, and HCC development in murine models of MASH [217].

Mechanistically, the protective effect is mediated by hepatic activation of PPARα and induction of PCK1, resulting in enhanced fatty acid oxidation, reduced aberrant glycolysis, and attenuation of chronic inflammation [217].

Nevertheless, although numerous mouse models have been described, many fail to faithfully reproduce the human disease, and only a minority reliably progress to HCC while providing a robust mechanistic link between MASLD and hepatocarcinogenesis [218]. Contrary to preclinical research, while observational studies in humans cannot fully elucidate the complex pathophysiological mechanisms underlying MASLD, they provide robust and long-standing evidence that, in patients with MASLD, the concomitant presence of T2DM is associated with a substantial increase in the risk of liver-related events, particularly HCC development [11].

Based on the integration of this basic and clinical evidence, a growing body of real-world findings has confirmed that targeting IR and associated metabolic alterations in MASLD-T2DM may translate into oncological benefits. Importantly, randomized studies and multicenter retrospective analyses have shown that the use of antidiabetic drugs—particularly metformin and other insulin-sensitizing agents—is associated with a reduced risk of HCC and improved survival in patients with T2DM [219]. Beyond its glucose-lowering effects [220], metformin exerts relevant pleiotropic actions on the gut microbiota [221], the gut–liver axis, and key metabolic and inflammatory pathways [222,223], contributing to a potential oncoprotective effect in HCC [224].

In parallel, other classes of antidiabetic agents—including Sodium–Glucose Cotransporter 2 (SGLT2) inhibitors [225,226,227], Dipeptidyl Peptidase-4 (DPP4) inhibitors [228], and Glucagon-Like Peptide-1 (GLP-1) receptor agonists [229,230]—are emerging as potential complementary tools in the management of MASLD–T2DM patients, owing to their favorable effects on body weight, inflammation, hepatic steatosis, and fibrogenesis.

Although evidence regarding their direct impact on HCC incidence and progression remains preliminary and partly controversial, available data suggest a potential clinical benefit, coupled with a generally favorable safety profile when appropriately monitored [230,231]. Overall, the rational repurposing of antidiabetic therapies represents a promising integrated strategy to improve HCC prevention and management in the setting of MASLD, reinforcing the paradigm of a metabolically informed and personalized approach to hepatic oncology. However, although targeting IR represents a rational and widely adopted therapeutic approach in MASLD and T2DM, its impact on HCC prevention and progression appears to be only partial and context dependent [232]. In particular, the insulin-sensitizing-based strategies primarily exert indirect antitumor effects by improving metabolic homeostasis and reducing systemic inflammation, without fully counteracting the complex network of oncogenic, inflammatory, and immunological pathways involved in hepatocarcinogenesis [233]. Moreover, interindividual variability in treatment response further highlights the need for more comprehensive and integrative therapeutic strategies [232,233].

In this sense, T2DM is associated with a marked reduction in progression-free and overall survival, even among patients undergoing curative or non-curative treatments, underscoring the central role of the metabolic–inflammatory axis in shaping the biological aggressiveness of the disease [234]. In this context, elucidating the pathogenetic mechanisms linking IR, chronic inflammation, and hepatic carcinogenesis represents a critical prerequisite for the identification of novel therapeutic targets and the development of personalized treatment strategies [219]. Although targeting IR remains a key component of metabolic intervention in MASLD–T2DM-associated HCC, growing evidence suggests that broader strategies addressing gut–liver axis dysregulation may be required to effectively modulate the complex immunometabolic mechanisms underlying hepatocarcinogenesis.

In this sense, among metabolism-targeted approaches, GLP-1 receptor agonists (GLP-1RAs) deserve particular attention because of their dual relevance in T2DM and MASLD and relative pleiotropic effects [235,236]. Beyond improving glycemic control, body weight, and IR, GLP-1RAs have shown beneficial effects on hepatic steatosis, inflammation, and cardiometabolic risk [235]. In this context, semaglutide has recently gained regulatory approval for the treatment of MASH, further supporting the clinical relevance of this drug class in metabolic liver disease [237,238]. Importantly, accumulating evidence suggests that GLP-1RAs may also influence hepatocarcinogenesis through indirect mechanisms, including reduction of chronic inflammation, improvement of lipotoxicity, and modulation of the hepatic immune microenvironment [239]. In parallel, emerging studies indicate that GLP-1RAs can reshape gut microbiota composition, increasing beneficial microbial taxa, improving intestinal barrier integrity, and reducing endotoxemia, thereby potentially restoring gut–liver axis homeostasis [240]. These pleiotropic effects make GLP-1RAs particularly attractive candidates within integrated strategies aimed at reducing liver disease progression and HCC risk in patients with MASLD and T2DM [236]. Future studies should clarify whether the benefits of GLP-1RAs on gut microbiota composition and immunometabolic pathways may translate into measurable reductions in HCC incidence or improved responses to systemic therapies, including immune checkpoint inhibitors. From this perspective, integrating GLP-1RAs into multimodal prevention and treatment strategies may represent a promising avenue for precision medicine in patients with MASLD and T2DM.

### 4.2. Is It Time to Enlarge Perspectives by Integrating Strategies Modulating the Gut–Liver Axis?

The complexity of HCC management requires innovative and personalized strategies, and within this scenario, the gut–liver axis emerges as a pathogenetic and therapeutic determinant of increasing relevance, with translational implications capable of reshaping the clinical approach to HCC.

The biological rationale supporting the targeting of the gut–liver axis in HCC is grounded in the observation that most liver tumors arise in the setting of chronic liver disease and cirrhosis, both conditions characterized by profound alterations in the intestinal microbiota, increased mucosal barrier permeability, and systemic bacterial translocation. Recently, Dolapchiev et al. underscored the role of the intestinal microbiome in modulating susceptibility to HCC in murine models of metabolic dysfunction-associated steatohepatitis (MASH) [241]. Through multi-omics analyses, the authors demonstrated that specific microbial signatures associated with metabolic endotoxemia activate pro-inflammatory and pro-oncogenic hepatic transcriptomic programs, further reinforcing the gut–liver axis as an amplifier of the pathogenic link between T2DM, MASLD, and HCC.

From a translational perspective, one of the most promising aspects lies in the possibility of modulating the intestinal microbiota to interfere with pro-tumorigenic signals originating from the gut. Preclinical models have demonstrated that selective microbiota depletion or compositional manipulation reduces HCC incidence and progression by attenuating hepatic inflammation [242,243,244].

In this sense, therapeutic strategies such as prebiotics, probiotics, and small molecules derived from natural products have shown promise in modulating the gut–liver axis, and immunomodulatory therapies targeting the gut–liver axis, representing a highly promising therapeutic strategy in HCC.

#### 4.2.1. Role of Probiotics, Prebiotics, and Natural Compounds

Recently, a growing body of preclinical and clinical evidence has consolidated the role of probiotics as emerging therapeutic tools in HCC, through modulation of the intestinal microbiota, microbial metabolites, and immune responses along the gut–liver axis. Experimental studies have shown that specific bacterial strains can enhance the efficacy of oncological therapies. In particular, *Akkermansia muciniphila* has been associated with improved response to anti–PD-1 therapy in HCC, mediated by remodeling of bile acid metabolism and the hepatic immune microenvironment [245].

Similarly, combined interventions with *Clostridium butyricum* and soluble dietary fiber have been shown to improve hepatic metabolic alterations via inhibition of the pro-inflammatory Acly/Nrf2/NF-κB pathways, suggesting a potential role in preventing progression toward HCC [246].

A central contribution of probiotics also emerges in the regulation of innate immunity: microbial production of butyrate is essential for the maturation and activation of liver-resident natural killer cells through the butyrate–IL-18 axis, with relevance in both physiological and pathological contexts [247,248]. On the adaptive immunity side, *Lactobacillus* species can modulate follicular helper T cells via the gut microbiota [249], while specific immunological indices, such as the Tfr/Tfh ratio, have been proposed as predictors of HCC recurrence [250]. Moreover, *Bifidobacterium pseudolongum* and its metabolite acetate have demonstrated a direct suppressive effect on MASLD-associated HCC, reinforcing the role of microbial metabolites as antitumor mediators [251]. Collectively, these studies indicate that probiotics represent a biologically grounded and potentially customizable strategy to influence HCC progression and optimize therapeutic response.

However, the modulation of gut microbiota represents a double-edged sword. While strategies aimed at restoring microbial balance may enhance immune responses and therapeutic efficacy, external factors capable of disrupting microbiota composition may exert the opposite effect [252,253].

In particular, antibiotic exposure has been consistently associated with reduced efficacy of ICIs, likely due to depletion of beneficial microbial taxa involved in immune activation and maintenance of antitumor immunity [253]. This observation highlights the critical role of microbiota integrity in determining treatment outcomes and suggests that careful consideration of antibiotic use in patients undergoing immunotherapy [206,253].

In this context, bioactive compounds of natural origin are also emerging as multitarget modulators capable of interfering with intestinal dysbiosis, barrier permeability, and liver-derived inflammatory signaling. Qu et al. demonstrated that ginsenoside Rk3 suppresses hepatic carcinogenesis through profound remodeling of the intestinal microbiota, reducing bacterial translocation and attenuating hepatic inflammation, thus highlighting a direct involvement of the gut–liver axis in tumor growth control [254]. Consistently, Jing et al. showed that an *Echinacea purpurea* polysaccharide inhibits HCC progression by modulating gut microbiota composition and suppressing TLR4/NF-κB pathway activation, thereby reducing chronic inflammation and tumor proliferation [255]. Also, studies focusing on intestinal barrier function further reinforce this paradigm. Chen et al. demonstrated that red rice seed coat ameliorates alcoholic liver disease by restoring intestinal integrity and normalizing the gut microbiota through inhibition of Sphingosine Kinase 2 (SPHK2), a mechanism also relevant in the prevention of hepatic carcinogenesis [256]. Similarly, Ram et al. showed that nimbolide attenuates gut dysbiosis, prevents bacterial translocation, and reduces hepatic inflammation in HCC models, primarily by strengthening intestinal barrier integrity [257].

In this evolving scenario, fecal microbiota transplantation (FMT) has also emerged as a potential microbiota-directed strategy [258]. By transferring a healthy donor microbial ecosystem, FMT may restore intestinal eubiosis, improve barrier integrity, reduce endotoxemia, and modulate systemic and hepatic immune responses [259,260]. Although evidence in MASLD-related HCC remains preliminary, early clinical and preclinical data suggest that FMT may improve metabolic parameters, attenuate hepatic inflammation, and potentially enhance responsiveness to systemic therapies, including ICIs [261]. Further studies are required to define its long-term safety, optimal donor selection, and therapeutic positioning within personalized treatment algorithms [258].

In parallel with this modern opening frontier, the development of small molecules capable of interfering with specific immunoregulatory pathways activated along the gut–liver axis, including FXR-mediated signaling, has shown encouraging results in experimental models and clinical studies.

#### 4.2.2. Bile Acid Signaling and FXR-Targeted Strategies: Translational Opportunities

Increasing evidence positions bile acids as central metabolic and signaling hubs at the interface of the gut–liver axis, with significant implications for the clinical management of HCC. Large-scale prospective data from the European Prospective Investigation into Cancer and Nutrition (EPIC) cohort demonstrate that distinct alterations in circulating bile acid profiles are detectable several years before HCC diagnosis, supporting a contributory role of bile acid dysregulation in early hepatocarcinogenesis [262].

Beyond their pathogenic role, selected bile acids exert hepatoprotective and immunomodulatory effects with substantial translational relevance. Among these, ursodeoxycholic acid (UDCA) has been extensively investigated and clinically used in the management of cholestatic and chronic liver diseases due to its cytoprotective properties and ability to regulate bile acid homeostasis. Recent evidence suggests that UDCA favorably modulates the gut–liver axis by activating the FXR/FGF15 signaling pathway, regulating apoptosis and autophagy, improving intestinal barrier integrity, and mitigating liver injury driven by microbial-associated molecular patterns (MAMPs) [263].

Moreover, UDCA has been reported to exert antitumor effects in murine HCC models by enhancing CD8^+^ T cell function and suppressing tumor growth through remodeling of the bile acid pool within the tumor microenvironment [264]. In this scenario, beyond their diagnostic relevance, bile acids thus emerge as functional regulators of the tumor microenvironment and immune landscape, representing targets for therapeutic intervention. Recent evidence has further expanded interest in FXR agonists as potential therapeutic tools in HCC and related metabolic liver diseases. In particular, obeticholic acid (OCA), a potent FXR agonist, has been shown to improve MASLD-associated phenotypes through selective remodeling of the intestinal microbiota—characterized by increased abundance of beneficial bacteria such as *Akkermansia muciniphila*, *Bifidobacterium* spp., and *Bacteroides* spp.—and by regulation of bile acid synthesis and serum levels [265,266,267].

Comprehensively, these findings support the integration of bile acid profiling and bile acid-centered interventions into precision medicine approaches aimed at improving early detection, prognostic assessment, and personalized treatment strategies in HCC.

In parallel, increasing attention is being devoted to natural compounds capable of modulating FXR activity. In murine models of liver cancer, celastrol has shown significant antitumor effects, associated with reduced relative abundance of *Bacteroides fragilis*, increased glycine conjugation of bile acids, and inhibition of oncogenic pathways through regulation of the FXR/RXRα–mTOR axis [268]. Collectively, these data reinforce FXR as a crucial regulatory node of the gut–liver axis and a promising therapeutic target in HCC. From a clinical perspective, integrating the gut–liver axis into HCC management may enable a transition from a “one-size-fits-all” approach toward truly personalized medicine. Although prospective clinical studies are required to validate these strategies, current evidence clearly indicates that the gut–liver axis represents not only a central pathogenetic mechanism but also a high-impact translational therapeutic target, capable of improving efficacy, safety, and personalization of care in HCC.

Table 1 summarizes the most promising potential strategies targeting the gut–liver axis in MASLD-T2DM-related HCC treatment (Table 1).

#### 4.2.3. Role of Lifestyle Interventions and Bariatric Surgery

Beyond pharmacological approaches, lifestyle interventions remain a cornerstone strategy for reducing the burden of MASLD, T2DM, and their related hepatic complications. Dietary modification, regular physical activity, weight reduction, and smoking cessation may simultaneously target multiple pathogenic drivers involved in hepatocarcinogenesis, including insulin resistance, chronic inflammation, oxidative stress, and gut microbiota dysbiosis [269]. In particular, adherence to healthy dietary patterns such as the Mediterranean diet has been associated with improved metabolic control, reduced hepatic steatosis, and favorable modulation of gut microbiota composition [270]. Similarly, regular exercise improves insulin sensitivity, decreases visceral adiposity, and attenuates systemic inflammation, thereby potentially reducing liver disease progression and HCC risk [271]. Smoking cessation should also be emphasized, given the established association between tobacco exposure, oxidative stress, and cancer risk [272].

In selected patients with obesity and advanced MD, bariatric surgery represents an effective intervention with profound metabolic and hepatic benefits [273]. Sustained weight loss after bariatric procedures has been associated with improvement or resolution of steatohepatitis, regression of fibrosis, lower incidence of T2DM-related complications, and reduced long-term risk of major liver outcomes, including HCC [274]. In addition to weight reduction, bariatric surgery may exert beneficial effects through hormonal remodeling, improvement of IR, and reshaping of the gut microbiota [275,276].

Collectively, these observations support the integration of structured lifestyle interventions and metabolic surgery into multidisciplinary strategies aimed at preventing disease progression and hepatocarcinogenesis in high-risk patients.

### 4.3. Knowledge Gaps and Future Research Directions

The gut–liver axis represents a highly complex bidirectional communication system, mediated by dynamic chemical, metabolic, and immunological interactions between the intestinal microbiota and the liver. The evidence synthesized in this review delineates a central role for the gut microbiota and its metabolites—including LPS, bile acids, and SCFAs—in HCC progression and in the modulation of therapeutic responses. Nevertheless, despite rapid advances in this field, the molecular and immunological mechanisms governing these interactions remain largely incompletely defined [277].

A major limitation of the current literature is that most available evidence derives from animal models, which, although indispensable for mechanistic insights, do not fully recapitulate the biological complexity and evolutionary trajectory of human HCC, particularly in the context of MASLD and its close interconnections with T2DM and metabolic syndrome [278]. Similarly, microbiota-targeted interventions—including probiotics and small-molecule compounds—have shown encouraging results in preclinical settings; however, their translation into clinical practice remains constrained by the lack of robust evidence and large-scale prospective clinical trials [279].

In this context, the integration of multi-omics approaches—encompassing metagenomics, metabolomics, and transcriptomics—with advanced artificial intelligence (AI) tools, together with the exploitation of innovative platforms such as organoids, may partially bridge this knowledge gap. These strategies hold the potential to decode the complexity of the gut–liver axis, identify predictive biomarkers, and develop risk stratification models, thereby enabling precision medicine approaches [280]. Such integration could significantly improve the personalized management of MASLD-related HCC and open new avenues for rational, gut–liver axis-based therapeutic interventions.

Recent studies underscore the pivotal role of integrated multi-omics approaches in deciphering the complexity of the gut–liver axis in HCC and in identifying novel therapeutic targets.

Chen et al. employed a combined analysis of gut microbiota composition, bile acid profiling, and hepatic signaling, demonstrating that *Lactobacillus brevis* alleviates both HCC and T2DM through coordinated modulation of intestinal dysbiosis, bile acid metabolism, and the NOTCH1 pathway, revealing systemic pathogenic connections that are difficult to capture using single-dimensional approaches [281]. Similarly, Deng et al. integrated metabolomic, immunological, and microbiota data to show that XYXD enhances natural killer T (NKT) cell-mediated immunity by promoting primary bile acid synthesis and improving gut microbiota composition, resulting in synergistic antitumor effects [282]. Finally, Jing et al. combined microbiota profiling with inflammatory signaling analyses, demonstrating that *Echinacea purpurea* polysaccharide inhibits HCC progression by modulating the microbiota–TLR4/NF-κB axis [255].

Organoid-based models represent one of the most advanced experimental platforms for the study of liver diseases, offering a highly physiological three-dimensional and functional recapitulation of human tissues. In particular, hepatic organoids have demonstrated substantial potential for investigating the pathophysiological mechanisms underlying chronic liver diseases, including MASLD/MASH, providing a more representative experimental system than conventional two-dimensional cultures [283].

In parallel, the integration of organoids with microfluidic technologies has led to the development of organoids/organs-on-a-chip systems, enabling dynamic modeling of the gut–liver axis by reproducing physiological flows, metabolic gradients, and inter-organ interactions [284,285]. Within this framework, gut–liver-on-chip models allow controlled investigation of microbiota-derived metabolites on hepatocyte functions, including glucose and lipid metabolism, bile acid synthesis, and albumin and urea secretion [286]. These platforms are particularly relevant for HCC research, as they enable the analysis of dysbiosis-induced metabolic and immunological alterations in a human-relevant context.

Nevertheless, significant limitations persist, including the incomplete reproduction of the tumor microenvironment and complex intercellular signaling networks observed in vivo, underscoring the need for further refinement of these models for drug screening and precision medicine applications.

Conclusively, bridging current knowledge gaps will require the integration of advanced experimental models, multi-omics technologies, and prospective clinical investigations. Such efforts will be essential to translate the rapidly expanding understanding of gut–liver axis biology into clinically actionable strategies, paving the way toward more precise prevention, early detection, and personalized treatment of MASLD–T2DM-associated HCC. In this context, there is a pressing need to develop refined, risk-based surveillance strategies that integrate metabolic, inflammatory, and molecular determinants of carcinogenesis. Clinical variables including diabetes mellitus, obesity, and components of metabolic syndrome should be systematically incorporated into risk stratification models, alongside emerging biomarkers reflecting systemic inflammation, IR, and gut–liver axis dysfunction [6,7]. Furthermore, the recognition of MASLD as a risk factor not only for HCC but also for cholangiocarcinoma suggests that future surveillance approaches may need to broaden their scope beyond a single tumor entity. This expanded oncological spectrum reinforces the importance of identifying shared pathogenic pathways and developing integrated screening frameworks capable of capturing multiple liver cancer phenotypes [6,7].

From a translational perspective, advances in omics technologies, liquid biopsy, and microbiome profiling may provide novel tools for early cancer detection and individualized risk prediction. In particular, the characterization of gut microbiota signatures and their metabolites may offer innovative, non-invasive biomarkers reflecting the immunometabolic landscape underlying hepatocarcinogenesis.

Ultimately, a paradigm shift toward precision-based surveillance—grounded in the systemic nature of MD—will be essential to improve early diagnosis and reduce mortality in MASLD-related liver cancer [287,288,289,290,291,292,293,294,295,296,297,298,299].

## 5. Conclusions

HCC arising in the setting of MASLD and T2DM reflects a complex interplay of metabolic, inflammatory, and immunological mechanisms. While IR has long been considered the central pathogenic driver linking these conditions, growing evidence highlights the gut–liver axis as a crucial upstream regulator of hepatic immunometabolic homeostasis.

Collectively, these insights support a paradigm shift toward a more integrated view of hepatocarcinogenesis in MASLD–T2DM, in which targeting metabolic dysfunction and gut–liver axis alterations may represent promising complementary strategies for HCC prevention and management.

## Figures and Tables

**Table 1:** Potential therapeutic strategies targeting the gut–liver axis in MASLD–T2DM-associated hepatocellular carcinoma.

Therapeutic Category	Representative Interventions	Target Within the Gut–Liver Axis	Main Biological Effects	Potential Implications for HCC	References
**Microbiota-targeted therapies**	*Akkermansia* *muciniphila*, *Clostridium* *butyricum*, *Lactobacillus* spp., *Bifidobacterium pseudolongum*	Intestinal microbiota composition	Restoration of microbial diversity, SCFA production, modulation of bile acid metabolism, regulation of adaptive and innate immune responses.	Improved antitumor immune responses and potential enhancement of immunotherapy efficacy.	[254,255]
**Prebiotics and dietary modulation**	Soluble dietary fiber	Microbial fermentation pathways	Increased production of SCFAs, inhibition of inflammatory signaling (Acly/Nrf2/NF-κB pathways), improvement of metabolic homeostasis.	Reduction of hepatic inflammation and prevention of inflammation-driven hepatocarcinogenesis.	[247,254,258,259]
**Natural microbiota-modulating compounds**	Ginsenoside Rk3, *Echinacea purpurea* polysaccharide	Gut microbiota and inflammatory signaling	Remodeling of microbial composition, inhibition of TLR4/NF-κB signaling, reduction of bacterial translocation.	Attenuation of hepatic inflammation and suppression of tumor-promoting pathways.	[258,259]
**Barrier-protective interventions**	Nimbolide, red rice seed coat extract	Intestinal epithelial barrier integrity	Strengthening of tight junctions, inhibition of SPHK2 signaling, reduction of gut permeability and endotoxemia.	Decreased hepatic inflammatory activation and prevention of tumor progression.	[260,261]
**Metabolite-targeted strategies**	Microbial metabolites (butyrate, acetate)	Gut-derived immuno-metabolites	Regulation of NK cell maturation (butyrate–IL-18 axis), modulation of T cell responses and immune homeostasis.	Enhancement of antitumor immune surveillance.	[178,193,194,263]
**Bile acid-** **modulating** **therapies**	Ursodeoxycholic acid (UDCA)	Bile acid pool and gut–liver signaling	Activation of FXR/FGF15 pathway, regulation of autophagy and apoptosis, improvement of intestinal barrier function.	Suppression of tumor growth and modulation of tumor immune microenvironment.	[267,268]
**Host signaling-** **targeted therapies**	Obeticholic acid (FXR agonist), celastrol	FXR/RXRα signaling pathways	Regulation of bile acid synthesis, modulation of microbiota composition, inhibition of oncogenic pathways (mTOR signaling).	Potential disease-modifying strategy and personalized therapeutic intervention.	[269,270,271,272]

MASLD: Metabolic dysfunction-associated steatotic liver disease. T2DM: Type 2 diabetes mellitus. HCC: Hepatocellular carcinoma. SCFAs: Short-chain fatty acids. NK: Natural killer. IL: Interleukin. TLR4: Toll-like receptor 4. NF-κB: Nuclear factor kappa B. SPHK2: Sphingosine kinase 2. FXR: Farnesoid X receptor. FGF15: Fibroblast growth factor 15. RXRα: Retinoid X receptor alpha. mTOR: Mammalian target of rapamycin.

## Data Availability

No new data were created or analyzed in this study. Data sharing does not apply to this article.

## Abbreviations

The following abbreviations are used in this manuscript:

AGEs	Advanced Glycation End-products
AhR	Aryl Hydrocarbon Receptor
AKT	Protein Kinase B
AMPK	AMP-Activated Protein Kinase
ARNT	Aryl Hydrocarbon Nuclear Translocator
BAs	Bile Acids
BC	Bacillus Calmette–Guérin
BSH	Bile Salt Hydrolase
CAFs	Cancer-Associated Fibroblasts
CD	Cluster of Differentiation
CML	Nε-(Carboxymethyl)lysine
CRP	C-Reactive Protein
CSCs	Cancer Stem Cells
DAMPs	Damage-Associated Molecular Patterns
DCs	Dendritic Cells
DCA	Deoxycholic Acid
DPP4	Dipeptidyl Pepti-dase-4
DREs	Dioxin Response Elements
ECM	Extracellular Matrix
EGFR	Epidermal Growth Factor Receptor
ERK	Extracellular Signal-Regulated Kinase
EPIC	European Prospective Investigation into Cancer and Nutrition
FFAR2	Free Fatty Acid Receptor 2
FFAR3	Free Fatty Acid Receptor 3
FFAs	Free Fatty Acids
FGF15	Fibroblast Growth Factor 15
FXR	Farnesoid X Receptor
GLP-1	Glucagon-Like Peptide-1
GPR41	G-Protein-Coupled Receptor 41
GPR43	G-Protein-Coupled Receptor 43
GVB	Gut Vascular Barrier
HCC	Hepatocellular Carcinoma
HDACs	Histone Deacetylases
HFD	High-Fat Diet
HIF-1α	Hypoxia-Inducible Factor-1 Alpha
HMGB1	High Mobility Group Box 1
HSCs	Hepatic Stellate Cells
IDO1	Indoleamine 2,3-Dioxygenase 1
IGF-1	Insulin-Like Growth Factor 1
IGF-1R	Insulin-Like Growth Factor 1 Receptor
IGFBPs	Insulin-Like Growth Factor Binding Proteins
IKKβ	IκB Kinase Beta
IL	Interleukin
ILC	Innate Lymphoid Cell
IR	Insulin Resistance
IRS-1	Insulin Receptor Substrate-1
JAK	Janus Kinase
LCA	Lithocholic Acid
LPS	Lipopolysaccharide
MARCO	Macrophage Receptor with Collagenous Structure
MAPK	Mitogen-Activated Protein Kinase
MASLD	Metabolic Dysfunction-Associated Steatotic Liver Disease
MASH	Metabolic Dysfunction-Associated Steatohepatitis
MAMPs	Microbe-Associated Molecular Patterns
MCTs	Monocarboxylate Transporters
MD	Metabolic Dysfunction
MDSCs	Myeloid-Derived Suppressor Cells
mTOR	Mammalian Target of Rapamycin
NF-κB	Nuclear Factor Kappa B
NKT	Natural Killer T Cells
NLRP3	NOD-Like Receptor Family Pyrin Domain Containing 3
OCA	Obeticholic Acid
PAMPs	Pathogen-Associated Molecular Patterns
PAI-1	Plasminogen Activator Inhibitor 1
PCK1	Phosphoenolpyruvate Carboxykinase 1
PD-1	Programmed Cell Death Protein 1
PD-L1	Programmed Death Ligand 1
PI3K	Phosphatidylinositol 3-Kinase
PKA	Protein Kinase A
PPARα	Peroxisome Proliferator-Activated Receptor Alpha
PPP	Picropodophyllin
PRRs	Pattern Recognition Receptors
RAGE	Receptor for Advanced Glycation End-products
ROS	Reactive Oxygen Species
SCFAs	Short-Chain Fatty Acids
SGLT2	Sodium–Glucose Cotransporter 2
SIRT5	Sirtuin 5
STAT3	Signal Transducer and Activator of Transcription 3
STING	Stimulator of Interferon Genes
T2DM	Type 2 Diabetes Mellitus
TAGE	Toxic Advanced Glycation End-products
TDO2	Tryptophan 2,3-Dioxygenase
TGF-β	Transforming Growth Factor Beta
TIM-3	T cell Immunoglobulin and Mucin-domain Containing-3
TLR	Toll-Like Receptor
TNF-α	Tumor Necrosis Factor Alpha
Tregs	Regulatory T Cells
TGR5	Takeda G-Protein Coupled Receptor 5
UDCA	Ursodeoxycholic Acid
VEGF	Vascular Endothelial Growth Factor
YAP	Yes-Associated Protein
ZO	Zonula Occludens
